# Plant-Mediated Zinc Oxide Nanoparticles: Advances in the New Millennium towards Understanding Their Therapeutic Role in Biomedical Applications

**DOI:** 10.3390/pharmaceutics13101662

**Published:** 2021-10-11

**Authors:** Mahadevamurthy Murali, Nataraj Kalegowda, Hittanahallikoppal G. Gowtham, Mohammad Azam Ansari, Mohammad N. Alomary, Saad Alghamdi, Natarajamurthy Shilpa, Sudarshana B. Singh, M. C. Thriveni, Mohammed Aiyaz, Nataraju Angaswamy, Nanjaiah Lakshmidevi, Syed F. Adil, Mohammad R. Hatshan, Kestur Nagaraj Amruthesh

**Affiliations:** 1 Applied Plant Pathology Laboratory, Department of Studies in Botany, University of Mysore, Manasagangotri, Mysuru 570006, Karnataka, India; botany.murali@gmail.com (M.M.); knataraj922@gmail.com (N.K.); 2 Department of Studies in Biotechnology, University of Mysore, Manasagangotri, Mysuru 570006, Karnataka, India; gajendramurthygowtham@gmail.com (H.G.G.); shilpanataraj@gmail.com (N.S.); brijeshrajput.bt@gmail.com (S.B.S.); reachaiyaz@gmail.com (M.A.); 3 Department of Epidemic Disease Research, Institutes for Research and Medical Consultations (IRMC), Imam Abdulrahman Bin Faisal University, Dammam 31441, Saudi Arabia; 4 National Center for Biotechnology, Life Science and Environmental Research Institute, King Abdulaziz City for Science and Technology, P.O. Box 6086, Riyadh 11442, Saudi Arabia; malomary@kacst.edu.sa; 5 Laboratory Medicine Department, Faculty of Applied Medical Sciences, Umm Al-Qura University, Makkah P.O. Box 715, Saudi Arabia; ssalghamdi@uqu.edu.sa; 6 Department of Studies in Microbiology, University of Mysore, Manasagangotri, Mysuru 570006, Karnataka, India; lakshmiavina@rediffmail.com; 7 Central Sericultural Germplasm Resources Centre, Central Silk Board, Ministry of Textiles, Thally Road, TVS Nagar, Hosur 635109, Tamil Nadu, India; thrivenimc@gmail.com; 8 Department of Biochemistry, Karnataka State Open University, Mukthagangotri, Mysuru 570006, Karnataka, India; natabiochem@gmail.com; 9 Department of Chemistry, College of Science, King Saud University, Riyadh 11451, Saudi Arabia; sfadil@ksu.edu.sa (S.F.A.); mhatshan@ksu.edu.sa (M.R.H.)

**Keywords:** antimicrobial, antioxidant, antidiabetic, anticancer, anti-inflammatory, drug delivery, photocatalytic

## Abstract

Zinc oxide nanoparticles have become one of the most popular metal oxide nanoparticles and recently emerged as a promising potential candidate in the fields of optical, electrical, food packaging, and biomedical applications due to their biocompatibility, low toxicity, and low cost. They have a role in cell apoptosis, as they trigger excessive reactive oxygen species (ROS) formation and release zinc ions (Zn^2+^) that induce cell death. The zinc oxide nanoparticles synthesized using the plant extracts appear to be simple, safer, sustainable, and more environmentally friendly compared to the physical and chemical routes. These biosynthesized nanoparticles possess strong biological activities and are in use for various biological applications in several industries. Initially, the present review discusses the synthesis and recent advances of zinc oxide nanoparticles from plant sources (such as leaves, stems, bark, roots, rhizomes, fruits, flowers, and seeds) and their biomedical applications (such as antimicrobial, antioxidant, antidiabetic, anticancer, anti-inflammatory, photocatalytic, wound healing, and drug delivery), followed by their mechanisms of action involved in detail. This review also covers the drug delivery application of plant-mediated zinc oxide nanoparticles, focusing on the drug-loading mechanism, stimuli-responsive controlled release, and therapeutic effect. Finally, the future direction of these synthesized zinc oxide nanoparticles’ research and applications are discussed.

## 1. Introduction

Nanotechnology is a branch of science concerned with the processing and modification of materials at the nanometer scale. It is a product of nature that influences a material through physiochemical processes, and it has a wide range of applications in research [1,2,3,4]. At Caltech, Richard Feynman, the greatest theoretical physicist, was the first to communicate the idea of nanotechnology in 1959. Nanomaterials are microscopic materials with nanometer dimensions that are in use in different fields. They have distinct properties, such as a large surface area and quantum size effects, and are regarded as a significant state of matter [5]. Metal and metal oxide nanoparticles have many promising applications in agriculture, catalysts, electronics, fiber optics, sensors, biolabeling, and biomedical fields [6,7,8,9,10,11]. In the synthesis of nanoparticles, chemical methods often result in dangerous byproducts, and the chemical reducing reagents (such as hydrazine, sodium borohydride, and sodium dodecyl sulfate) used are toxic to nature. Hence, in comparison to the chemical methods, the eco-friendly green synthesis method has gained significant attention in the past decade for the synthesis of nanoparticles [4,8,12].

The synthesis of nanoparticles through biogenic methods involves the use of a variety of natural resources that include all forms of plants and microorganisms. During the synthesis of nanoparticles by biological route, active enzymes/biomolecules (active metabolites/phytochemicals) of the biotic source act as reducing and capping agents, thereby allowing for the mass production of the particles [1,13,14]. The usage of biotic sources not only reduces the risk of toxicity of the obtained particles to the environment but can also be produced in a large scale with the required parameters (well-defined size and morphology) [2,15,16]. Among the various metal nanoparticles synthesized through the usage of various biotic sources, zinc oxide nanoparticles synthesized through plant extracts have gained considerable importance in the present millennium due to their unique properties and various biomedical applications.

## 2. Zinc Oxide Nanoparticles

Zinc oxide is a unique inorganic material with semiconducting behavior and piezoelectric, triboelectric, pyroelectric, optoelectronics, and catalysts properties [12,17,18,19,20]. Zinc oxide is an n-type semiconductor with a band gap of 3.4 eV and an excitation binding energy of 60 meV at room temperature. Zinc oxide is nontoxic and skin-friendly, and it is even used as a UV blocker in sunscreens. Among the metal oxide nanoparticles, zinc oxide nanoparticles have been extensively used in various biological applications due to their nontoxic nature, and they are also listed as “generally recognized as safe” (GRAS) by the U.S. FDA (21CFR182.8991). Various studies have established zinc oxide as the most effective antimicrobial agent, as evidenced by releasing reactive oxygen species (ROS) on its surface [17].

Meanwhile, zinc oxide is known to be biocompatible and safe, with various applications in biomedical and drug delivery systems. Among the metal oxide nanoparticles, zinc oxide nanoparticles have piqued the interest of researchers for the past two decades due to their unique and wide range of properties. Due to their biocompatible nature, they are widely used as a semiconductor nanomaterial in ethanol gas sensors, photocatalysis, electronic and optoelectronic devices, pharmaceutical and cosmetic products, and, particularly, in the biomedicine field [10,20,21]. It has been well-documented that zinc oxide nanoparticles can be synthesized by different methods using various types of biotic sources, but the present review mainly focuses on the biosynthesis of zinc oxide nanoparticles using plant sources that are termed to be eco-friendly, cost-effective, and, also, efficient in biomedical applications, along with advances made in the “new millennium” towards understanding their role/mechanism of action involved.

## 3. Synthesis of Zinc Oxide Nanoparticles from Plants

Green synthesis is an emerging area in which nontoxic chemicals are generated using environmentally friendly and bio-safe reagents and may also be considered as a viable alternative to the physical and chemical methods. However, the main problems with green synthesis are nanoparticle stability and a lack of understanding of its mechanisms. Researchers are focusing their efforts on the green synthesis of metal and metal oxide nanoparticles from various biological sources such as plants, bacteria, fungi, yeast, algae, etc. [22,23,24,25]. Among the different sources, plants are considered to be vastly available and are known to possess phytoconstituents that are reported to be beneficial in various ways to humankind [26,27,28,29]. Plant parts such as leaves, stems, bark, roots, rhizomes, fruits, flowers, and seeds have been extensively used for the synthesis of zinc oxide nanoparticles in the recent past and are found to be stable, highly pure, cost-effective, and possess greater biomedical properties [30,31]. Plants are the most popular choice for nanoparticle synthesis, because they produce large quantities of stable, varied-sized nanoparticles for specific applications on a large scale [20,32,33]. Plant phytochemicals such as polysaccharides, proteins, amino acids, alkaloids, flavonoids, and terpenoids are used as stabilizing agents in green synthesis methods to biologically reduce metal oxides (or metal ions) to metal nanoparticles in an aqueous solution [5,25,34].

The green synthesis of zinc oxide nanoparticles from plant sources usually begins with the washing of plant parts using double-distilled water or Tween 20, accompanied by drying at room temperature and grinding into powder with a mortar and pestle. The plant extract is prepared by boiling the weighted powder with continuous stirring with a magnetic stirrer. The solution is then filtered through Whatman filter paper and used as a plant extract in the following steps. Zinc precursors such as zinc acetate [Zn(OAc)_2_], zinc chloride [ZnCl_2_], zinc nitrate [Zn(NO_3_)_2_] and zinc sulphate [ZnSO_4_] solutions are individually used with a fixed amount of plant extract, and it serves as the source for preparing zinc oxide nanoparticles through the application of various methods that involve heating, centrifugation, etc. that finally results in the formation of the desired nanoparticles [35,36,37,38,39,40]. Further, UV–Vis spectroscopy was used to confirm the nanoparticles that were synthesized. The morphology of the nanoparticles was determined using electron microscopes such as the Scanning Electron Microscope (SEM), Transmission Electron Microscope (TEM), and Atomic Force Microscopy (AFM). The nanoparticles’ crystal structure and chemical composition were calculated using X-Ray Diffraction (XRD), Energy Dispersive X-Ray Spectroscopy (EDS or EDX) used for determination of the elements present, and Fourier-transform infrared (FTIR) spectroscopy used to describe the functional groups present on the surfaces of the nanoparticles. The Dynamic Light Scattering (DLS) and Zeta Potential (ZP) methods were used to determine the size and dispersion of the nanoparticles in a liquid suspension.

It is well-documented that the ability of any organism to reduce metal ions apart from stabilizing them into nanoparticles is the basis for green synthesis. Plants are considered to be the best candidates for the green synthesis of nanoparticles, as they produce stable forms of the same compared to microorganisms [41]. Plants are considered to possess a rich biodiversity of secondary metabolites that are worth investigating. In the recent past, researchers were interested in the phytoconstituents produced by plants to investigate their bio-reduction process of metal nanoparticles by combinations of phytoconstituents/secondary metabolites [42]. These molecules not only acted as reducing agents but also played a key role in the capping of nanoparticles, which was crucial for their stability and biocompatibility, and due to these molecules, no extra chemical reducing and capping agents were required [4]. In addition, these plant-based molecules not only operated as the growth terminator of zinc oxide nanoparticles but also acted as a linker molecule between two or more molecules of zinc oxide-formed ZnO NPs, making them self-assemble [43].

Although many reports are available on the bioactivities (such as antimicrobial, antioxidant, anticancer, photocatalytic, etc.) of zinc oxide nanoparticles synthesized from various plant species, a few studies have been conducted to compare the activities of nanoparticles from different plants [12,44,45,46]. In addition, it is also noted that the plant-mediated zinc oxide nanoparticles possessed better biological activities than the nanoparticles synthesized through the chemical route [12,28,44,47,48]. Further, it has been noted that, due to the coating of various pharmacologically active biomolecules on their surface, zinc oxide nanoparticles allow multiple ligand-based conjugations of nanoparticles with their respective receptors, leading to better bioactivities [3,4,12,14,45,46,47,49,50]. These comparisons have suggested that zinc oxide nanoparticles possess better bioactivities when they are synthesized from the potential reducing agents present in the plants. Table 1 lists the different plant sources used in zinc oxide nanoparticles synthesis, along with their morphological characteristics and applications.

## 4. Biomedical Applications of Plant-Mediated Zinc Oxide Nanoparticles

### 4.1. Antibacterial Activity

**Figure 1:**
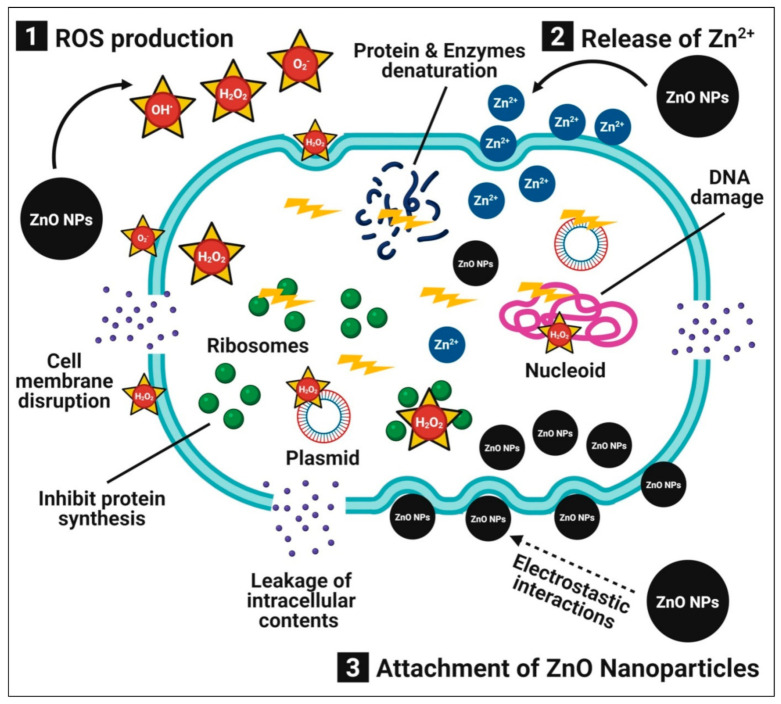
Possible antibacterial mechanisms of plant-mediated zinc oxide nanoparticles.

Bacterial infections are a major threat to humanity’s health. Researchers have focused on metal and metal oxide nanoparticles as antibacterial agents due to the increased antibiotic resistance in bacteria and the emergence of new strains [2]. Zinc oxide nanoparticles have been studied extensively as antibacterial agents due to their unique physiochemical properties and increased surface areas [4]. Furthermore, zinc oxide nanoparticles are both safe and compatible with the human body. Numerous publications describe the possible antibacterial mechanisms of zinc oxide nanoparticles, which involves (1) the production of ROS (i.e., OH^•^ (hydroxyl radical) and O_2_^−2^ (peroxide)), which induces oxidative stress, cell membrane disruption, and DNA damage, resulting in the death of bacterial cells; (2) the dissolution of zinc oxide nanoparticles into the release of Zn^2+^ ions, which interact with the bacterial cell, especially the cell membrane, cytoplasm, and nucleic acid, thus disintegrating the cellular integrity and resulting in bacterial cell death; and (3) direct interactions between zinc oxide nanoparticles and bacterial cell membranes through electrostatic forces that damage the plasma membrane and cause a leakage of intracellular components [30] (Figure 1).

In our previous study, zinc oxide nanoparticles synthesized from leaf extracts of *Ceropegia candelabrum* with zinc nitrate (as a precursor) by the hydrothermal process resulted in nanoparticles of high purity, a hexagonal wurtzite shape, and 12–35-nm sizes [5]. The biosynthesized zinc oxide nanoparticles significantly showed antibacterial potential against *Staphylococcus aureus*, *Bacillus subtilis*, *Escherichia coli*, and *Salmonella typhi* at 100 μg·mL^−1^. One of our research groups also bio-fabricated zinc oxide nanoparticles from leaf extracts of *Cochlospermum religiosum* to study their antibacterial potentiality [82]. The bio-fabricated zinc oxide nanoparticles significantly exhibited the inhibition of Gram-positive (*B. subtilis* and *S. aureus*) and Gram-negative (*Pseudomonas aeruginosa* and *E. coli*) bacteria with minimum inhibitory concentrations (MIC) of 4.8–312.5 μg·mL^−1^. The antibacterial potency of zinc oxide nanoparticles is probably due to their small size, which is more likely to damage the membrane, penetrate the cytoplasm, and produce numerous ROS that can damage the DNA and other cellular components of bacteria [31,80].

The difference in antibacterial activity can be associated with the structural and chemical compositions of the bacterial cell membranes [2]. Sharma et al. [127] studied the effect of the shape and size of zinc oxide nanoparticles synthesized by using *Aloe vera* leaf extracts on their antibacterial activity and reported that cuboidal (40–45 nm)-shaped nanoparticles were shown to be more protuberant in antibacterial activity when compared to spherical (60–180 nm) and hexagonal (~65 nm)-shaped nanoparticles. Due to the small size of biosynthesized zinc oxide nanoparticles, they provide a high surface area, which leads to more interactions between nanoparticles and bacterial cells, which can be used as an antibacterial agent even at lower dosages. Biosynthesized zinc oxide nanoparticles initially interact with the bacterial plasma membrane, causing nanoparticles to enter the cytoplasm and release metal ions, thereby disrupting the membrane permeability and, finally, causing DNA damage, leading to the death of bacterial cells [71]. The antibacterial activity demonstrated by zinc oxide nanoparticles synthesized from leaf extracts of *Cassia fistula* and *Melia azadarach* have a substantial ability to suppress clinical pathogens (such as *E. coli* and *S. aureus*) compared to traditional drugs, and it was concluded that the synthesis of nanoparticles employing the extracts of medicinal plants could be effective in the treatment of various human infectious diseases [126]. Recently, Faisal et al. [11] showed that 1% zinc oxide nanoparticles (1 mg mL^−1^) synthesized from aqueous fruit extracts of *Myristica fragrans* were found to display the maximum zone of inhibition against *Klebsiella pneumoniae*, *E. coli*, *P. aeruginosa*, and *S. aureus*. Further, the antibacterial efficacy of plant-mediated zinc oxide nanoparticles against various bacterial pathogens is listed in Table 2.

### 4.2. Antifungal Activity

**Figure 2:**
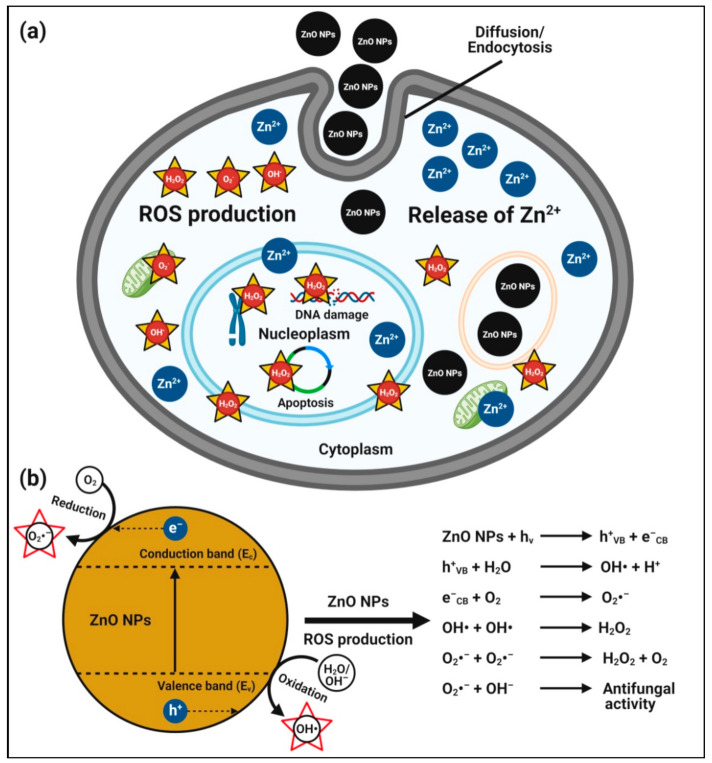
Possible antifungal mechanism of plant-mediated zinc oxide nanoparticles. (**a**) Hypothetical anticandidal mechanisms of zinc oxide nanoparticles, and (**b**) plausible mechanisms of action of zinc oxide nanoparticle-induced ROS generation for their anticandidal activity.

In addition to antibacterial activity, zinc oxide nanoparticles also possess antifungal activity against many harmful fungi and yeasts, making them promising antifungal food additives [19,103]. The possible mechanisms of antifungal activity of zinc oxide nanoparticles are described for zinc oxide nanoparticles that can enter fungal (conidial) cells by diffusion and endocytosis; they interfere in the mitochondrial function and promote ROS production Zn^2+^ ion release inside the cytoplasm. The excess production of ROS and Zn^2+^ ions released from zinc oxide nanoparticles can penetrate the nuclear membrane and cause irreversible DNA damage, inducing cell death [134,135] (Figure 2). The role of the electronic band gap (E_g_) property of zinc oxide nanoparticles with the redox potential (EH) of numerous ROS generation reactions have also been proposed [134]. Zinc oxide nanoparticles electrons (e^−^), when excited with energy higher than E_g_, are promoted across the band gap energy to the conduction band edge (E_c_), which creates a hole (h^+^) in the valence band (E_v_). The e^−^ in E_c_ and holes in E_v_ exhibit high reducing and oxidizing power, respectively. The e^−^ reacts with molecular oxygen (O_2_) to produce superoxide anion (O_2_^•–^) through sequential reduction reactions. The h^+^ can extract e^−^ from water (H_2_O) and/or hydroxyl ions to generate hydroxyl radical (OH^•^).

The zinc oxide nanoparticles synthesized from flower extracts of *Nyctanthes arbor-tristis* demonstrated antifungal activity against fungal phytopathogens (such as *Fusarium oxysporum*, *Botrytis cinerea*, *Penicillium expansum*, *Alternaria alternata*, and *Aspergillus niger*) with the lowest MIC value of 16 μg·mL^−1^, suggesting that they could be used to develop antifungal agents for commercial use in agriculture [32]. Similarly, Khan et al. [39] suggested that zinc oxide nanoparticles synthesized using *Trianthema portulacastrum* extracts were found to exhibit antifungal properties against *Aspergillus niger*, *A. flavus*, and *A. fumigatus*. Additionally, zinc oxide nanoparticles synthesized from different plant extracts such as *Beta vulgaris*, *Cinnamomum tamala*, *C. verum*, and *Brassica oleracea* were found effective against *Candida albicans* and *A. niger* [25]. Furthermore, the plant-mediated zinc oxide nanoparticles that were effective antifungal agents against various fungal pathogens are provided in Table 2.

### 4.3. Antioxidant Activity

**Figure 3:**
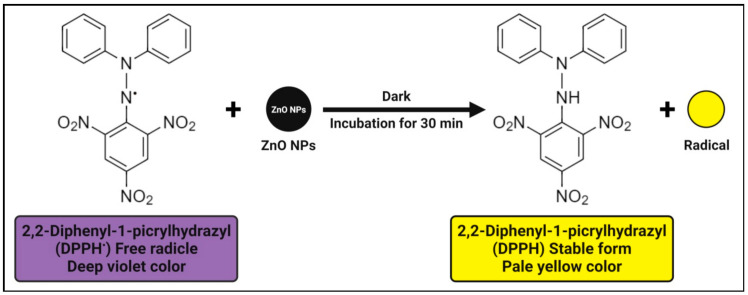
Possible antioxidant mechanisms of plant-mediated zinc oxide nanoparticles.

The antioxidant activity of zinc oxide nanoparticles is attributed to their smaller size, but another explanation may be a phenomenon in 2,2-diphenyl-1-picrylhydrazyl (DPPH) where the electron density is transferred from the oxygen atom to the odd electron located at the nitrogen atom, resulting in a decrease in the n → π* transition intensity at 517 nm. The antioxidant activity’s mechanism involves the unstable deep violet-colored methanolic solution of DPPH, which turns into stable pale-yellow color with the addition of zinc oxide nanoparticles due to their DPPH free radical scavenging activity through the transfer of an electron from the oxygen atom to the odd electron of a nitrogen atom, resulting in stable DPPH molecule formation (Figure 3) [83]. In other words, the antioxidant property of zinc oxide nanoparticles is determined by their ability to donate hydrogen. Furthermore, even in the absence of UV light, the formation of a large number of electron and hole pairs on the surfaces of zinc oxide nanoparticles results in a high redox potential that splits water (H_2_O) molecules into hydroxyl (OH^•^) and hydrogen (H^•^) radicals, which are available for DPPH free radical reduction and DPPH molecule stability [17,83,124].

Our previous study reported that the antioxidant activity of biosynthesized zinc oxide nanoparticles from *C. candelabrum* showed significantly DPPH free radical scavenging activity from 0% to 55%, with an IC_50_ value of 95.09 μg·mL^−1^, compared with the 75% inhibition offered by ascorbic acid (a positive control) at 50 μg·mL^−1^ [5]. It is also reported that an increased antioxidant activity was observed with an increase in the zinc oxide nanoparticle concentration. According to the findings of Khan et al. [39], the green synthesized zinc oxide nanoparticles had a strong antioxidant activity due to their charge density and capping materials on their surface. Alamdari et al. [83] revealed that zinc oxide nanoparticles biosynthesized from leaf extracts of *Sambucus ebulus* were found to exhibit H_2_O_2_free radical scavenging activity with an IC_50_ value of 43 μg·mL^−1^ and concluded that the presence of metal or Zn ions in the structure could enhance the antioxidant capabilities of the biosynthesized zinc oxide nanoparticles. Recently, Faisal et al. [11] revealed that zinc oxide nanoparticles synthesized from the aqueous fruit extracts of *M. fragrans* were found to show excellent free radical scavenging irrespective of the methods employed, viz., ABTS (82.12% TEAC), DPPH (66.3% FRSA), TAC (71.1 μg AAE·mg^−1^), and TRP (63.41 μg AAE·mg^−1^). In accordance with the above findings, the antioxidant efficacy of plant-mediated zinc oxide nanoparticles is represented in Table 3.

### 4.4. Antidiabetic Activity

**Figure 4:**
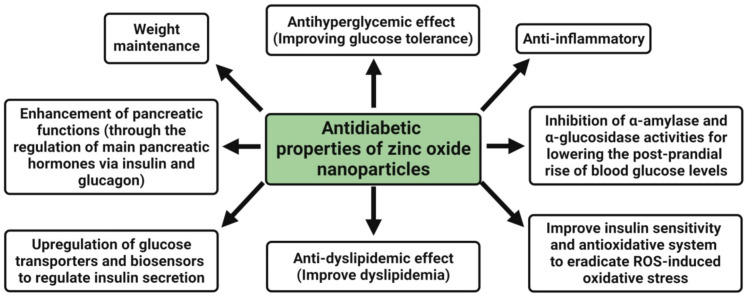
Possible antidiabetic mechanism of plant-mediated zinc oxide nanoparticles.

Diabetes is a complex disease that requires an effective multifaceted treatment approach to care for patients. Antidiabetic medications are often used in conjunction with insulin or other drugs, resulting in higher medication costs overall. Zinc supplementation has been shown to improve glycemic regulation in diabetic humans and animals [33,66]. Furthermore, this metal can boost diabetic problems such as nephropathy and cardiomyopathy [35]. Due to their capacity to deliver Zn^2+^ ions, zinc oxide nanoparticles are currently being studied to treat diabetes and diabetic complications. The antidiabetic properties of zinc oxide nanoparticles are an antihyperglycemic effect, i.e., improving the glucose tolerance, enhancement of pancreatic functions (through the regulation of main pancreatic hormones, namely insulin and glucagon), upregulation of glucose transporters and biosensors to regulate insulin secretion, weight maintenance, anti-dyslipidemic effect (i.e., improve dyslipidemia), anti-inflammatory, inhibition of α-amylase and α-glucosidase activities for lowering the post-prandial rise of blood glucose levels, and improving the insulin sensitivity and antioxidative system to eradicate ROS-induced oxidative stress [138] (Figure 4).

The inhibition of carbohydrate metabolizing enzymes (such as α-amylase and α-glucosidase) is a unique strategy for controlling blood sugar, and it requires a large number of compounds to be evaluated in order to rule out inactive molecules and save time and money. In vitro α-amylase and α-glucosidase inhibitory studies revealed that zinc oxide nanoparticles synthesized from *Tamarindus indica* extract had the highest inhibitory activity compared to the zinc oxide nanoparticles synthesized from other plant extracts [22]. Additionally, Rajakumar et al. [34] confirmed that the synthesized zinc oxide nanoparticles from *Andrographis paniculata* leaf extract displayed better antidiabetic potential in terms of α-amylase inhibition activity with aIC_50_ value of 121.42 μg·mL^−1^. Similarly, the zinc oxide nanoparticles synthesized from *Costus igneus* leaf extract were found to show more effective antidiabetic activity, wherein the percentage of inhibition ranged from 20% to 74% for α-amylase and 36% to 82% for α-glucosidase at aconcentration of 20–100 μg·mL^−1^ of nanoparticles [33]. Recently, Faisal et al. [11] indicated that an excellent α-amylase (73.23%) and α-glucosidase (65.21%) inhibition activity at 400 μg·mL^−1^ was offered by zinc oxide nanoparticles synthesized from aqueous fruit extracts of *M. fragrans* and revealed that the bio-based nanoparticles could have strong antidiabetic properties and are considered a useful therapeutic agent for the treatment of diabetes as a replacement for expensive and ineffective medicines. Additionally, other reports on the efficacy of antidiabetic activity by plant-mediated zinc oxide nanoparticles are listed in Table 4.

### 4.5. Anticancer Activity

The therapeutic regimes cannot distinguish between cancerous and normal cells, resulting in systemic toxicity and side effects. As a result, new chemotherapeutic agents with high selectivity for cancer cells are needed. Zinc deficiency initiates and facilitates the development of cancerous cells, as it is an important mineral that maintains homeostasis by controlling the enzyme activities in our body [104]. Zinc is essential for the function of p-53 (a tumor suppressor gene) that controls apoptosis by activating the Caspase-6 (Casp6) enzyme. In addition, zinc oxide nanoparticles have a special electrostatic property that aids in the selective targeting of cancer cells. Anionic phospholipids are abundant on the surface of cancer cells, resulting in electrostatic attraction with zinc oxide nanoparticles, which encourages cancer cells to take up zinc oxide nanoparticles, resulting in cytotoxicity in cancer cells [70,129].

**Figure 5:**
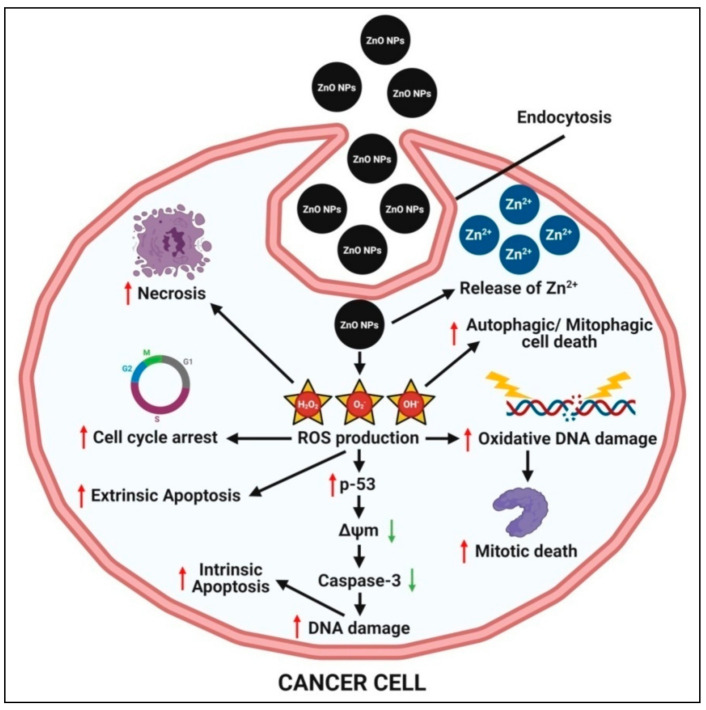
Possible anticancer mechanism of plant-mediated zinc oxide nanoparticles.

The small size of zinc oxide nanoparticles, on the other hand, aids in the permeation and retention of nanoparticles within tumorous cells, allowing them to act. The possible mechanisms behind zinc oxide nanoparticles’ selective pH-responsive cytotoxicity towards cancer cells are (1) the pH-dependent rapid dissolution of zinc oxide nanoparticles into the release of Zn^2+^ ions under an acidic intracellular environment, which causes oxidative stress (via ROS production) and subsequent cell damage within cancer cells, and (2) the production of a large amount of ROS in cancer cells relative to normal cells; the elevated ROS level then causes mitochondrial dysfunction and activates the intrinsic mitochondrial apoptotic pathway (Figure 5) (Sana et al. [71]. ROS can also mediate cell death via extrinsic necrosis and apoptosis. Further, the overproduction of ROS leads to induced oxidative DNA damage and mitotic death and can also trigger autophagic/mitophagic cell death [19,26]. The MTT assay has been effectively used to confirm the anticancer activity of zinc oxide nanoparticles prepared using the plant-mediated green synthesis method against a variety of cell lines, which are detailed in Table 5. In addition, zinc oxide nanoparticles have been used successfully as an effective carrier to deliver anticancer drugs to tumor cells.

Zinc oxide nanoparticles have a particle size, shape, surface charge, concentration, and time-dependent cytotoxicity in cancer cells, and photo-irradiation with ultraviolet (UV) or near-infrared (NIR) lasers enhance its anticancer activity through a synergistic chemo-photodynamic effect [29,71]. The majority of the researchers also demonstrated that zinc oxide nanoparticles are less or nontoxic compared to normal cells in vitro when used in the same concentration range as cancer cells. Zinc oxide nanoparticles can cause systemic toxicity in tumor-bearing mouse models. However, using antioxidants in combination with zinc oxide nanoparticles may minimize the toxic side effects and increase the antitumor potential [11]. Zinc oxide nanoparticles’ adverse toxic side effects can also be prevented or decreased by using cancer cell-specific ligands. While numerous reports on the anticancer properties of plant-mediated green synthesized zinc oxide nanoparticle in vitro systems are available, only a few studies in tumor-bearing mouse models have been performed [93]. In vivo tumor models can be used to conduct further research using plant-mediated green synthesized zinc oxide nanoparticles with active cancer cell-targeting strategies.

The semiconducting nature of biosynthesized zinc oxide nanoparticles has been reported to induce cytotoxicity in cancer cells by forming ROS on the particle’s surface; the released Zn^2+^ ions are dissolved in culture media, indicating the direct interaction of nanoparticles with a cancer cell membrane, resulting in oxidative stress and, ultimately, cancer cell death [71]. Zinc oxide nanoparticles synthesized from the aqueous extract of *Deverra tortuosa* exhibited enticing selective cytotoxic efficacy against two cancer lines (Caco-2 and A549), providing appealing ‘safer and cheaper’ alternatives to traditional therapy regimens [99]. Faisal et al. [11] used the *Streptomyces* 85E strain to elucidate the protein kinase inhibition capability of zinc oxide nanoparticles synthesized from aqueous fruit extracts of *M. fragrans* and concluded that the plant extracts provide crucial capping and stabilizing agents to biogenic nanoparticles, which are responsible for their anticancer properties. Recently, Umamaheswari et al. [29] reported that the anticancer activity of zinc oxide nanoparticles biosynthesized from leaf extracts of *Raphanus sativus* was higher after treating the A549 cell lines, indicating that cancer drugs can be prepared using this environmentally friendly method. The DNA damage pathways, paraptosis, autophagy, radio sensitizing, aberrant cellular metabolism, overcoming chemo resistance, oxidative stress modulation, induction of apoptosis, arresting of the cancer cell cycle, anti-invasion, and metastasis are some of the possible anticancer mechanisms that have been reported so far [139].

### 4.6. Anti-Inflammatory Activity

**Figure 6:**
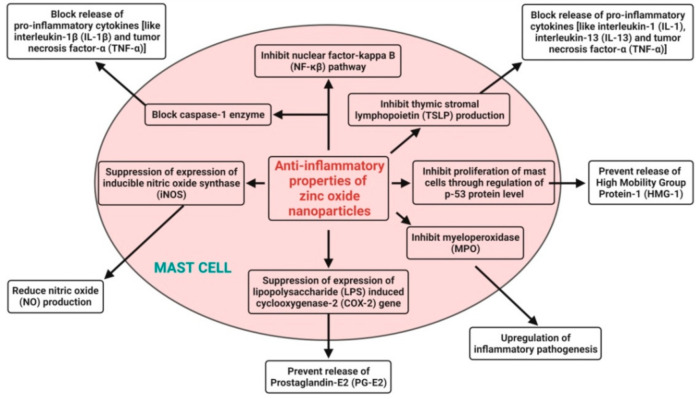
Possible anti-inflammatory mechanisms of plant-mediated zinc oxide nanoparticles.

Nanoparticles have been widely exploited as a potential anti-inflammatory agent in recent years. The large surface area-to-volume ratio causes nanoparticles to have more surface reactive properties, resulting in more interactions with the cell membrane and easier transport within the membrane [34,97,125]. Zn is easily transported through the cell membrane because of the nanosizes of zinc oxide nanoparticles. The anti-inflammatory mechanisms adopted by zinc oxide nanoparticles involve the release of Zn^2+^ ions from the dissolution of zinc oxide nanoparticles and subsequent block of the release of proinflammatory cytokines (like interleukin-1 (IL-1), interleukin-1β (IL-1β), interleukin-13 (IL-13), and tumor necrosis factor-α (TNF-α)) in mast cells; they suppress the expression of the lipopolysaccharide (LPS)-induced cyclooxygenase-2 (COX-2) gene in macrophages to prevent the release of prostaglandin-E2 (PG-E2), suppress the expression of inducible nitric oxide synthase (iNOS) to reduce nitric oxide (NO) production, inhibit myeloperoxidase (MPO) for the upregulation of inflammatory pathogenesis, inhibit the nuclear factor-kappa B (NF-κβ) pathway and block the caspase-1 enzyme in activated mast cells, inhibit the proliferation of mast cells through the regulation of the p-53 protein level to prevent the release of High Mobility Group Protein-1 (HMG-1), and also, inhibit thymic stromal lymphopoietin (TSLP) production by primary epithelial cells [140] (Figure 6).

The zinc oxide nanoparticles synthesized from *Polygala tenuifolia* root extract displayed excellent anti-inflammatory activity, even up to 1 mg·mL^−1^, by suppressing the LPS-induced mRNA and protein expressions of iNOS; COX-2; and anti-inflammatory cytokines (IL-1β, IL-6, and TNF-α) in LPS-stimulated RAW 264.7 murine macrophage cells in a dose-dependent manner [17]. Agarwal and Shanmugam [21] also revealed that green synthesized zinc oxide nanoparticles from *Kalanchoe pinnata* leaf extract were found to be biocompatible up to 1 mg·mL^−1^ and to have an anti-inflammatory effect by inhibiting the production and release of proinflammatory mediators such as IL-1β, IL-6, TNF-α, and COX-2. Likewise, Liu et al. [125] proved the anti-inflammatory effects of synthesized zinc oxide nanoparticles from *Vernonia amygdalina* leaf extract against different pain and inflammation-induced mice. Moreover, the synthesized zinc oxide nanoparticles were found to exhibit potent anti-inflammatory effects via reducing the inflammatory response and proinflammatory cytokines level in mice. Other reports on the anti-inflammatory properties of plant-mediated zinc oxide nanoparticles are summarized in Table 6.

### 4.7. Photocatalytic Activity

To prevent water pollution, various semiconductors, including zinc oxide, have been evaluated as photocatalysts and have proposed a viable solution. Zinc oxide nanoparticles synthesized from biological origin have shown a remarkable photocatalytic degradation of several dyes, including methylene blue, methyl orange, etc., due to their intrinsic ability to absorb UV irradiation and optical transparency. Since methylene blue is a major water pollutant emitted by industries such as textiles and is widely recognized as a traditional organic pollutant, these zinc oxide nanoparticles have gained much publicity due to environmental concerns [92,110,130]. Furthermore, the toxic effluents discharged by the textile industry have harmed marine life and the ecosystem, resulting in a serious environmental crisis. The bio-fabricated zinc oxide nanoparticles were utilized as a photocatalyst to degrade carcinogen organic dyes where the structure, particle size, crystallinity, photocatalyst band gap, surface area, and other factors were considered [40,96].

**Figure 7:**
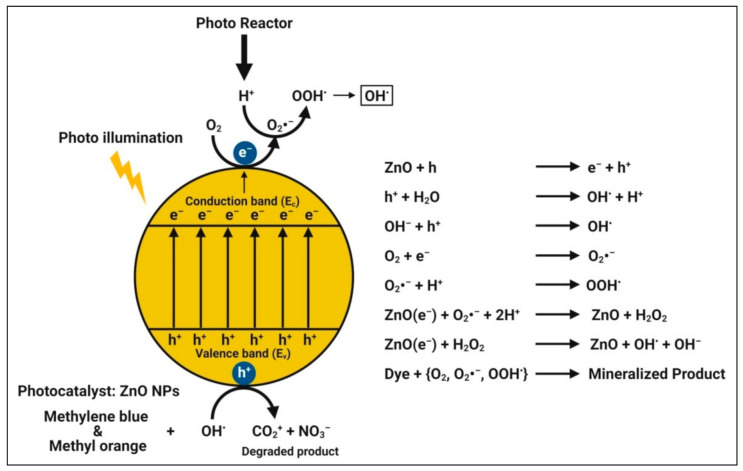
Possible photocatalytic mechanism of plant-mediated zinc oxide nanoparticles.

The mechanism of photodegradation is that, when semiconductors (catalysts) consume energy (photons) greater than their bandgap energy, charge separation occurs due to an electron jumping from the valence band to the conduction band of the catalyst, resulting in the creation of a hole in the valence band [64,70,108,122]. The created electron-hole pairs diffuse onto the photocatalyst’s surface and participate in the chemical reaction with the electron donor and acceptor when illuminated by light stronger than the band gap energy. With super strong oxidization, certain free electrons and holes convert the surrounding oxygen or water molecules into highly reactive unstable OH^•^ free radicals. When zinc oxide nanoparticles absorb photons with energies greater than their band gap energy from illumination, photocatalytic reactions begin to degrade the dyes [83] (Figure 7). Therefore, among the other zinc species in aqueous dye solutions under the experimental conditions, Zn^2+^ ions must be the most abundant, followed by Zn(OH)^+^, Zn(OH)_3_^−^, and Zn(OH)_4_^2–^.

In the presence of solar light as a catalyst, the biosynthesized zinc oxide nanoparticles showed a strong degradation capability for Synozol navy blue K-BF, and the hypothesized degradation mechanism yielded the formation of hydroxyl radical and dye degradation into smaller fragments [39]. Alamdari et al. [124] indicated that zinc oxide nanoparticles biosynthesized from the leaf extract of *S. ebulus* showed the maximum degradation of methylene blue dye noticeably around 80% after 200 min of UV radiation. Recently, Faisal et al. [11] reported that zinc oxide nanoparticles biosynthesized from aqueous fruit extracts of *M. fragrans* were used as photocatalytic agents, resulting in 88% of methylene blue dye degradation in 140 min of UV light illumination. In addition, Vinayagam et al. [130] reported that zinc oxide nanoparticles synthesized from *Bridelia retusa* leaf extract promoted the photocatalytic degradation of Rhodamine B dye by up to 94.74% within 165 min of solar irradiation. The crystalline and mesoporous natures, high surface area, and good electron acquiring features of the zinc oxide nanoparticles might be attributed to the accelerated degradation. Furthermore, the improved photocatalytic activity could be attributed to the stabilizing phytocompounds. Likewise, the photocatalytic activity of the plant-mediated zinc oxide nanoparticles is represented in Table 7.

### 4.8. Wound-Healing Activity

Since our skin is our body’s largest organ, it protects us from external attack, and any damage to it results in a wound. The healing of a wound takes time and can be slowed by microbial infection (*P. aeruginosa* and *S. aureus*). Nanoparticles have antibacterial properties and produce hydrogen peroxide, which causes cell damage [87]. Metal oxide nanoparticles can be utilized to destroy the pathogenic species and thereby improve wound healing [67]. Zinc oxide nanoparticles synthesized biologically from plant sources can be tremendously used as a wound-healing agent for treating general wounds and wounds caused due to burning [67,87,114]. Khatami et al. [114] indicated that zinc oxide nanoparticles (about 26 nm in size) synthesized from *Prosophis fracta* and coffee were found to impregnate on cotton wound bandages, conferring patches with a strong antimicrobial effect. Hence, they can potentially be used for treating and covering infection-sensitive wounds, namely diabetic or burns wounds. Shao et al. [87] biosynthesized zinc oxide nanoparticles from a *Barleria gibsoni* leaf extract, which acted as efficient and superior tropical antimicrobial formulations for the healing of burn infections by exhibiting a remarkable wound-healing potential in rats. Kiran Kumar et al. [142] studied zinc oxide nanoparticles synthesized from a *Raphanus sativus* root extract and showed their antimicrobial property against *Escherichia fergusonii* and *E. coli* strains that can be further explored for wound-healing potential. Apart from these, many reports have also indicated that the application of zinc oxide nanoparticles/or their biocomposites, irrespective of their application, possessed potential wound-healing ability and help in wound healing [143,144,145,146].

### 4.9. Targetted Drug Delivery System

**Figure 8:**
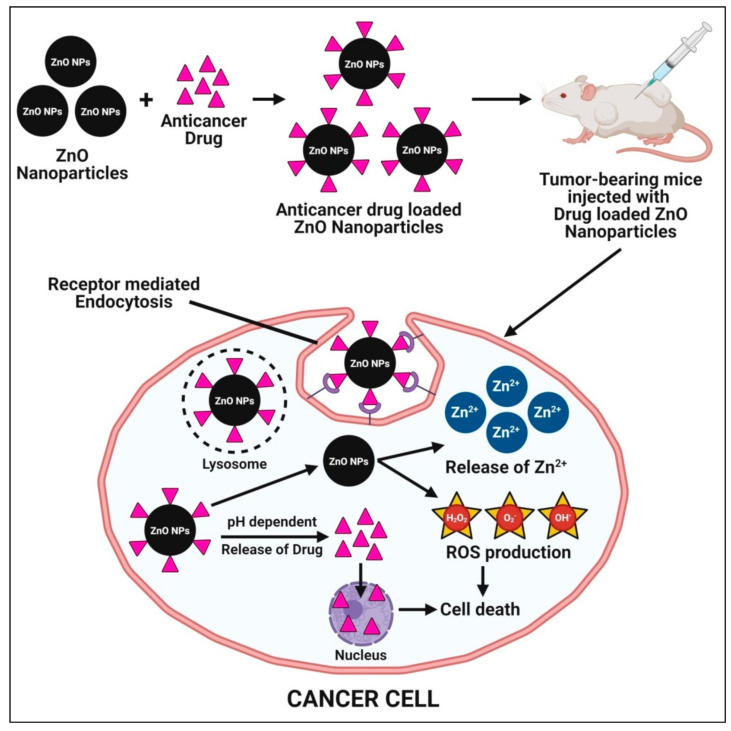
Possible mechanisms of plant-mediated zinc oxide nanoparticles in a targeted drug delivery system.

Zinc oxide nanoparticles have been considered as a potential candidate for targeted drug delivery because of their ease of synthesis from low-cost metal precursors, biocompatibility, and successful cellular internalization via endocytosis. Nanoparticles’ smaller particle sizes and larger surface areas make it easier for them to penetrate cell membranes and be absorbed into cells, resulting in better distribution. As a result, nanoparticles are commonly used in drug delivery systems, where they are loaded with drugs that reach their targets in appropriate quantities and then guide the drug release from the nanoparticles [51]. The effect of zinc oxide nanoparticles on drug delivery revealed that these nanoparticles aid in the rapid release of the drug at first and then transition to regulate the release over the treatment period [95]. The mechanisms underlying the enhanced cytotoxic potential of anticancer drug-loaded zinc oxide nanoparticles involve the pH-dependent release of targeted drug and zinc oxide nanoparticles in the cytoplasm once the drug-loaded zinc oxide nanoparticles enter through receptor-mediated endocytosis. The release of targeted anticancer drugs act upon cancer cells and cause cell death. Additionally, zinc oxide nanoparticles release Zn^2+^ ions and produce excessive ROS (oxidative stress) and, subsequently, lead to cancer cell death (Figure 8).

Vimala et al. [52] used green synthesized doxorubicin (DOX)-loaded zinc oxide nanoparticles with *Borassus flabellifer* fruit extract in a drug delivery system for anticancer therapy. The synthesized DOX–zinc oxide nanoparticles showed dose-dependent cytotoxicity against the human breast cancer MCF-7 and colon cancer HT-29 cell lines, with an inhibitory concentration (IC_50_) of 0.125 μg·mL^−1^. Additionally, the toxicity assessment in vivo showed that these DOX–zinc oxide nanoparticles had low systemic toxicity in the murine model system. The low toxicity and high anticancer therapy efficacy of DOX–zinc oxide nanoparticles were found to provide strong evidence for plant-mediated green synthesized zinc oxide nanoparticles as promising candidates in drug delivery systems. Akbarian et al. [95] successfully loaded an important anticancer drug (Paclitaxel, PTX) on chitosan-coated zinc oxide nanoparticles, which were synthesized from the ethanolic leaf extract of *Camellia sinensis* and evaluated their biological activity as a drug delivery system on breast cancer cell lines (MCF-7). The PTX loaded on the chitosan-coated zinc oxide nanoparticles were found to show acytotoxic (anticancer) effect on cancerous MCF-7 cell lines and reduced the cytotoxic effect on normal fibroblast cell lines. Despite the reports on the use of zinc oxide nanoparticles in targeted drug delivery systems, further research is required to develop zinc oxide nanoparticles synthesized from plant sources as a flexible drug delivery system for targeted delivery. Passive targeting administered nanoparticles preferentially accumulate in solid tumors with discontinuous endothelium based on the enhanced permeability and retention (EPR) phenomenon, which is directly dependent on the size of the particles, which is smaller than the tumor inter-endothelial gap cut-off size. Apart from this, the two fundamental characteristics of neoplastic tissues, such as leaky vasculature and impaired lymphatic drainage, may also facilitate nanoparticle targeting [147].

### 4.10. Tissue Engineering and Regenerative Medicine

Zinc oxide nanoparticles have emerged as promising materials for tissue engineering and regenerative medicine applications, which can help to promote tissue regeneration while reducing immune responses and preventing infections because of their antineoplastic, angiogenic, ultraviolet scattering, and wound-healing properties [148]. The osteogenesis and angiogenesis effects of zinc oxide nanoparticles have been effectively proven in several investigations due to their extraordinary antibacterial activity, and inexpensive cost [149,150]. Zinc oxide nanoparticles are also found to promote cell growth, proliferation, differentiation, and metabolic activity in a variety of cell lines for tissue engineering and regenerative medicine applications [151]. As a result, the use of plant-mediated zinc oxide nanoparticles in tissue engineering and regenerative medicine has become even more specialized and utilizing the innovative concept that zinc oxide nanoparticles’ proangiogenic properties might be tremendously advantageous in increasing the integration of advanced scaffolds into host tissue, as evidenced by in vitro and in vivo experiments.

Shubha et al. [152] synthesized zinc oxide nanoparticles using Gallic acid isolated from the aqueous extract of *Phyllanthus emblica* and reported that the smaller-sized plant-mediated zinc oxide nanoparticles were found to cause noticeably lesser toxicity than clinically advocated zinc oxide nanoparticles. Since they employed 3T3 fibroblasts from Balb mice, which are important connective tissue cells that aid in tissue repair and regeneration, and because these zinc oxide nanoparticles are benign to the cells, they can be used close to connective tissue cells. Shafique et al. [153] reported that biosynthesized zinc oxide nanoparticles from a *Cymbopogon citratus* leaf extract were found, showing a promising callogenesis and regeneration frequency at their optimum concentrations (20 mg·L^−1^ and 30 mg·L^−1^ in the internodes and seeds, respectively) in *Panicum virgatum*, and it was confidently believed that this finding would open new doors in tissue culture technology by increasing the frequency of plant in vitro regeneration in different plant groupings.

## 5. Future Perspective and Conclusion

Zinc oxide nanoparticles have superior properties in contrast to bulk materials due to their small size and large surface area-to-volume ratio, which makes them explored in various fields, viz., biomedical, biosensor, cosmetic, food industries, etc. Among the different types of zinc oxide nanoparticle synthesis, the nanoparticles synthesized by the green route using plant extract have gained considerable importance in the new millennium due to their eco-friendly, nontoxic, cost-effective nature that can also be reproduced under a large scale. The phytoconstituents/secondary metabolites of plant extracts readily reduce the metal ions and stabilize them into nanoparticles. These phytofabricated zinc oxide nanoparticles offer better antimicrobial, anticancer, anti-inflammatory, and antidiabetic, along with other biomedical, applications due to the coating of various pharmacologically active biomolecules on their surfaces. This review elucidated the eco-friendly nature of green synthesized zinc oxide nanoparticles and their potential application in various biomedical fields, including drug delivery systems, along with their mechanism of action involved at the cellular level. From this review, it may be noted that plant-mediated zinc oxide nanoparticles possess better biological properties that are of human importance and can also be used in drug delivery systems due to their biocompatibility. Inconclusively, this review discussed the possible mechanisms behind plant-mediated green synthesized zinc oxide nanoparticles and advances made towards their role in therapeutic applications in the new millennium.

## Figures and Tables

**Table 1:** Morphology of plant-mediated zinc oxide nanoparticles and their applications.

Plant Name	Plant Part Used	Size (nm)	Shape/ Morphology	Applications	Reference
**ZINC NITRATE**					
*Solanum nigrum*	Leaf	29	Quasi-spherical	Antibacterial	Ramesh et al. [51]
*Borassus flabellifer*	Fruit	55	Rod like	Drug delivery	Vimala et al. [52]
*Phyllanthus niruri*	Leaf	25	Quasi-spherical	Photocatalytic	Anbuvannan et al. [53]
*Anisochilus carnosus*	Leaf	30–40	Quasi-spherical	Antibacterial and Photocatalytic	Anbuvannan et al. [54]
*Hibiscus subdariffa*	Leaf	12–46	Spherical	Antibacterial and Anti-diabetic	Bala et al. [35]
*Plectranthus amboinicus*	Leaf	20–50	Spherical and hexagonal	Antibacterial, Antibiofilm and Larvicidal	Vijayakumar et al. [55]
*Carissa edulis*	Fruit	50–55	Flower shape	Photocatalytic	Fowsiya et al. [56]
*Rosa canina*	Fruit	<50	Spherical	Antibacterial, Antioxidant and Anticancer	Jafarirad et al. [57]
*Nephelium lappaceum*	Peel	25–40	Spherical	Photocatalytic	Karnan and Selvakumar [58]
*Limonia acidissima*	Leaf	12–53	Spherical	Antibacterial (TB)	Patil and Taranath [59]
*Carica papaya*	Milk latex	11–26	Hexagonal	Photocatalytic and Antibacterial	Sharma [60]
*Boswellia ovalifoliolata*	Bark	20	Spherical	Antimicrobial	Supraja et al. [61]
*Camellia sinensis*	Leaf	-	Hexagonal	Photocatalytic	Nava et al. [62]
*Ceropegia candelabrum*	Leaf	12–35	Hexagonal wurtzite	Antibacterial and Antioxidant	Murali et al. [5]
*Ziziphus nummularia*	Leaf	12–26	Spherical and irregular	Antifungal and Anticancer	Padalia and Chanda [7]
*Vaccinium arctostaphylos*	Fruit	13	Spherical	Anti-diabetic	Bayrami et al. [63]
*Citrus sinensis*	Peel	12–24	Hexagonal prisms and oval spheres; highly irregular sponge-like	Photocatalytic	Luque et al. [64]
*Mangifera indica*	Leaf	45–60	Nearly spherical and hexagonal quartzite	Antioxidant and Anticancer	Rajeshkumar et al. [65]
*Costus pictus*	Leaf	40	Hexagonal, rod-shaped and spherical	Antimicrobial and Anticancer	Suresh et al. [66]
*Solanum torvum*	Leaf	28	Spherical	Toxicological effect in Wistar albino rats	Ezealisiji et al. [67]
*Artabotrys hexapetalu*	Leaf	20–30	Spherical and rod-like	Antibacterial and Photocatalytic	Shanavas et al. [68]
*Bambusa vulgaris*					
*Annona squamosa*	Leaf	20–50	Hexagonal and quasi hexagonal plate like	Antibacterial and Anticancer	Ruddaraju et al. [69]
*Scutellaria baicalensis*	Root	33–99	Spherical	Antioxidant and Anticancer	Tettey and Shin [70]
*Albizia lebbeck*	Bark	66	Irregular spherical	Antibacterial, Antioxidant, Cytotoxic and Antiproliferative	Umar et al. [26]
*Citrus sinensis*	Peel	33	Hexagonal	Antibacterial, Antifungal and Anticancer	Gao et al. [19]
*Beta vulgaris*	Plant	20	Spherical	Antibacterial and Antifungal	Pillai et al. [25]
*Cinnamomum tamala*		30	Rod		
*Cinnamomum verum*		46	Spherical		
*Brassica oleracea* var. *italica*		47	Spherical		
*Crotalaria verrucosa*	Leaf	16–38	Hexagonal	Antibacterial and Anticancer	Sana et al. [71]
**ZINC NITRATE HEXAHYDRATE**					
*Azadirachta indica*	Leaf	40	Spherical	Antimicrobial	Elumalai and Velmurugan [13]
*Vitex trifolia*	Leaf	30	Nearly spherical and hexagonal	Antimicrobial and Photocatalytic	Elumalai et al. [72]
*Plectranthus amboinicus*	Leaf	88	Rod shape	Photocatalytic	Fu and Fu [73]
*Polygala tenuifolia*	Root	33–73	Spherical	Antioxidant and Anti-inflammatory	Nagajyothi et al. [17]
*Allium sativum* and *A. cepa*	Bulbs	14–70	Spherical	Photocatalytic activity	Stan et al. [74]
*Petroselinum crispum*	Leaf				
*Pongamia pinnata*	Leaf	100	Spherical, nanorod and hexagonal	Antibacterial	Sundrarajan et al. [75]
*Cassia fistula*	Leaf	~5–15	Sponge like irregular	Photocatalytic, Antioxidant and Antibacterial	Suresh et al. [76]
*Artocarpus gomezianus*	Fruit	11.53	Spherical	Photocatalytic and Antioxidant	Suresh et al. [77]
*Corymbia citriodora*	Leaf	64	Polyhedron	Photocatalytic	Zheng et al. [78]
*Azadirachta indica*	Leaf	10–30	Hexagonal	Antibacterial, Antioxidant and Photocatalytic	Madan et al. [36]
*Terminalia chebula*	Fruit	12	Roughly spherical	Photocatalytic	Rana et al. [79]
*Citrullus colocynthis*	Fruit	85–100	Flower	Antibacterial, Antioxidant and Anticancer	Azizi et al. [80]
	Seed	20–35	Hexagonal		
	Pulp	30–80	Irregular polygons		
*Ocimum tenuiflorum*	Leaf	10–20	Spherical	Non-enzymatic glucose sensor	Dayakar et al. [81]
*Cochlospermum religiosum*	Leaf	∼76	Hexagonal	Antibacterial and Antimitotic	Mahendra et al. [82]
*Azadirachta indica*, *Hibiscus rosa-sinensis*, *Murraya koenigii*, *Moringa oleifera* and *Tamarindus indica*	Leaf	27–54	Spherical	Antioxidant and Anti-diabetic	Rehana et al. [22]
*Eucalyptus globulus*	Leaf	11.6	Spherical	Antioxidant and Photocatalytic	Siripireddy and Mandal [83]
*Acacia senegal*	Arabic gum	10	Spherical	Photocatalytic	Taghavi Fardood et al. [84]
*Conyza canadensis*	Leaf	–	Irregular	Antibacterial and Photocatalytic	Ali et al. [85]
*Garcinia mangostana*	Fruit pericarp	21	Spherical	Photocatalytic	Aminuzzaman et al. [86]
*Andrographis paniculata*	Leaf	57	Spherical, oval and hexagonal	Antioxidant, Anti-diabetic and Anti-inflammatory	Rajakumar et al. [34]
*Barleria gibsoni*	Leaf	50	Hexagonal (Wurtzite)	Wound healing	Shao et al. [87]
*Anacardium occidentale*	Leaf	33	Hexagonal	Anticancer	Zhao et al. [88]
*Gracilaria edulis*	Aqueous	20–50	Hexagonal (Wurtzite) rod	Anticancer	Asik et al. [89]
*Populus ciliata*	Leaf	60–70	Sphere like	Antibacterial	Hafeez et al. [90]
*Mentha pulegium*	Leaf	40	Quasi- spherical	Antimicrobial	Rad et al. [91]
*Laurus nobilis*	Leaf	20–30	Spherical and hexagonal	Antibacterial and Photocatalytic	Chemingui et al. [92]
*Justicia wynaadensis*	Leaf	∼39	Hexagonal	Antimitotic and DNA-binding activities	Hemanth Kumar et al. [8]
*Artocarpus heterophyllus*	Leaf	12–24	Spherical	Anticancer	Majeed et al. [93]
*Eucalyptus globules*	Leaf	52–70	Spherical or globular	Antifungal	Ahmad et al. [94]
*Camellia sinensis*	Leaf	11	Sphere	Drug delivery	Akbarian et al. [95]
*Cinnamomum verum*	Bark	~45	Hexagonal wurtzite	Antibacterial	Ansari et al. [2]
*Ziziphus jujuba*	Fruit	29	Spherical	Photocatalytic	Golmohammadi et al. [96]
*Mussaenda frondosa*	Leaf	8–15	Hexagonal	Antibacterial, Antioxidant, Antidiabetic, Anticancer, Anti-inflammatory and Photocatalytic	Jayappa et al. [97]
	Stem	9–12	Spherical		
	Leaf-derived callus	5–7			
*Aegle marmelos*	Juice	~20	Hexagonal	Antibacterial, Antioxidant and Photocatalytic	Mallikarjunaswamy et al. [98]
*Zea mays*	Husk	300–550	Flower-like	Antibacterial and Antioxidant	Quek et al. [31]
*Artocarpus heterophyllus*	Peel	380–900	Cauliflower-like		
*Punica granatum*	Peel	260–500	Nanoflowers		
*Deverra tortuosa*	Plant	9–31	Hexagonal	Anticancer	Selim et al. [99]
**ZINC ACETATE**					
*Passiflora caerulea*	Leaf	70	Spherical	Antibacterial	Santhoshkumar et al. [100]
*Cucumis melo inodorus*	Rough shell	25–40	Crystals with pseudo spherical	Anticancer	Mahdizadeh et al. [101]
*Hyssops officinalis*	Plant	20–40	Pseudo spherical	Anti-angiogenesis, Anti-inflammatory and Anticancer	Rahimi Kalateh Shah Mohammad et al. [102]
*Syzgium cumini*	Seed	50–60	Spherical	Larvicidal	Roopan et al. [38]
*Lycopersicon esculentum*	Leaf	10–50	Hexagonal wurtzite	Antimicrobial and Anticancer	Vijayakumar et al. [103]
*Costus igneus*	Leaf	25–40	Hexagonal	Antibacterial, Antioxidant and Antidiabetic	Vinotha et al. [33]
*Rehmanniae radix*	Plant	10–12	Rod shape	Anticancer	Cheng et al. [104]
		∼200	Spherical		
*Cratoxylum formosum*	Leaf	∼500	Nanosheets	Antibacterial and Anticancer	Jevapatarakul et al. [105]
*Syzygium cumini*	Leaf	64–78	Spherical	Photocatalytic	Rafique et al. [106]
*Hyssopus officinalis*	Leaf	20–40	Pseudo spherical	Anticancer activity	Rahimi Kalateh Shah Mohammad et al. [107]
*Thlaspi arvense*	Plant	70–90	Flower	Antibacterial and Photocatalytic	Ullah et al. [108]
*Raphanus sativus*	Leaf	66	Spherical	Anticancer	Umamaheshwari et al. [29]
**ZINC ACETATE DIHYDRATE**					
*Anchusa italica*	Flower	~8–14	Hexagonal	Antimicrobial and Cytotoxicity	Azizi et al. [6]
*Lobelia leschenaultiana*	Leaf	20–65	Spherical and hexagonal	Acaricidal	Banumathi et al. [109]
*Mimosa pudica*	Leaf	27	Wurtzite and hexagonal	Photocatalytic	Fatimah et al. [110]
*Coffea arabioca*	Seed	46	Wurtzite and hexagonal	Photocatalytic	
*Pongamia pinnata*	Seed	30–40	Spherical	Anticancer and Antibiofilm	Malaikozhundan et al. [111]
*Couroupita guianensis*	Leaf	–	Hexagonal	Antibacterial	Sathishkumar et al. [112]
*Catharanthus roseus*	Leaf	50–92	Hexagonal wurtzite	Antibacterial	Gupta et al. [113]
*Nyctanthes arbor-tristis*	Flower	12–32	–	Antifungal	Jamdagni et al. [32]
*Coffea arabica*	Seeds	26	Spherical	Wound-healing	Khatami et al. [114]
*Ferulago angulata*	Boiss	32–36	Spheroid	Photocatalytic	Mehr et al. [115]
*Averrhoa bilimbi*	Fruit	37	Spherical	Photoelectrode	Ramanarayanan et al. [116]
*Coccinia abyssinica*	Tuber	10.4	Hexagonal	Antibacterial and Antioxidant	Safawo et al. [117]
*Atalantia monophylla*	Leaf	30	Spherical and hexagonal	Antimicrobial	Vijayakumar et al. [103]
*Kalanchoe pinnata*	Leaf	24	Hexagonal and spherical	Antioxidant, Anticancer and Anti-inflammatory	Agarwal and Shanmugam [21]
*Berberis aristata*	Leaf	20–40	Needle like	Antibacterial and Antioxidant	Chandra et al. [118]
*Juglans regia*	Leaf	45–65	Spherical	Antibacterial and Anticancer	Darvishi et al. [119]
		95–150	Flower		
*Cucurbita pepo*	Leaf	8	Spherical	Cytotoxicity	Hu et al. [120]
*Pandanus odorifer*	Leaf	90	Spherical	Antibacterial and Anticancer	Hussain et al. [121]
*Dolichos lablab*	Leaf	29	Hexagonal wurtzite	Bactericidal and Photocatalytic	Kahsay et al. [122]
*Abelmoschus esculentus*	Okra mucilage	29–70	Spherical, elongated, and rod-like	Photocatalytic	Prasad et al. [123]
*Musa acuminata*	Peel	30−80	Triangular-like	Photocatalytic	Abdullah et al. [40]
*Mucuna pruriens*	Seed	60	Flower and spherical	Antibacterial	Agarwal et al. [27]
*Sambucus ebulus*	Leaf	25−30	Spherical	Antibacterial, Antioxidant and Photocatalytic	Alamdari et al. [124]
*Vernonia amygdalina*	Leaf	20–40	Cylindrical	Anti-inflammatory	Liu et al. [125]
*Cassia fistula* and *Melia azadarach*	Leaf	3–68	Spherical	Antibacterial	Naseer et al. [126]
*Aloe vera*	Leaf	∼65	Hexagonal	Antibacterial and Photocatalytic	Sharma et al. [127]
		60–180	Spherical		
		40–45	Cuboidal and Rod		
*Calliandra haematocephala*	Leaf	19	Flower	Photocatalytic	Vinayagam et al. [128]
*Euphorbia fischeriana*	Root	30	Spherical	Anticancer	Zhang et al. [129]
*Myristica fragrans*	Fruit	43–83	Spherical or elliptical	Antibacterial, Antiparasitic, Antioxidant, Antidiabetic, Anticancer and Photocatalytic	Faisal et al. [11]
*Bridelia retusa*	Leaf	11	Flower-shape	Photocatalytic	Vinayagam et al. [130]
**ZINC SULPHATE**					
*Aloe barbadensis*	Leaf	8–18	Spherical, oval and hexagonal	Antibacterial	Ali et al. [131]
*Bauhinia tomentosa*	Leaf	22–94	Hexagonal	Antibacterial	Sharmila et al. [30]
*Trianthema portulacastrum*	Plant	25–90	Spherical	Antibacterial, Antifungal, Antioxidant, Anticancer and Photocatalytic	Khan et al. [39]
**OTHERS**					
*Tecoma castanifolia*	Leaf	70–75	Spherical	Antibacterial, Antioxidant, and Anticancer	Sharmila et al. [132]
*Trifolium pratense*	Flower	60–70	Agglomerated	Antibacterial	Dobrucka and Długaszewska [15]
*Jacaranda mimosifolia*	Flower	2–4	Spherical	Antibacterial	Sharma et al. [60]
*Heritiera fomes* and *Sonneratia apetala*	Bark and leaf	40–50	–	Antibacterial, Antioxidant, Anti-diabetic and Anti-inflammatory	Thatoi et al. [16]
*Sedum alfredii*	Shoots	100	Columnar in shape	Photocatalytic	Wang et al. [133]
*Juglans regia*	Leaf	–	–	Antifungal	Saemi et al. [134]
Plants	–	–	–	Antifungal	Sun et al. [135]

**Table 2:** Antibacterial and antifungal activity of plant-mediated zinc oxide nanoparticles.

Plant Name	Plant Part Used	Pathogen Name	Minimum Inhibitory Concentration (MIC) * (mg·mL^−1^)	Results	Reference
**ANTIBACTERIAL ACTIVITY**					
*Anisochilus carnosus*	Leaf	*Salmonella paratyphi*, *Vibrio cholerae*, *Staphylococcus aureus* and *Escherichia coli*	-	Showed antibacterial activity towards various human pathogens	Anbuvannan et al. [54]
*Hibiscus subdariffa*	Leaf	*Escherichia coli* and *Staphylococcus aureus*	0.05	Exerted better bactericidal property on *S. aureus* and *E. coli*	Bala et al. [35]
*Azadirachta indica*	Leaf	*Staphylococcus aureus*, *Pseudomonas aeruginosa*, *B. subtilis*, *Proteus mirabilis*, *E. coli*	0.006–0.05	Showed significant inhibition against bacterial strains in a dose-dependent manner	Elumalai and Velmurugan [13]
*Vitex trifolia*	Leaf	*Staphylococcus aureus*, *Bacillus subtilis*, *Pseudomonas aeruginosa*, *Proteus mirabilis* and *Escherichia coli*	0.006–0.05	Showed outstanding antibacterial activity against Gram positive and Gram negative bacteria	Elumalai et al. [72]
*Pongamia pinnata*	Leaf	*Staphylococcus aureus* and *Escherichia coli*	0.1	Superior antibacterial activity against Gram positive and Gram negative bacteria	Sundrarajan et al. [75]
*Cassia fistula*	Leaf	*Klebsiella aerogenes*, *Escherichia coli*, *Pseudomonas desmolyticum* and *Staphylococcus aureus*	0.5–0.1	Showed an excellent bactericidal activity against pathogenic bacteria	Suresh et al. [76]
*Plectranthus amboinicus*	Leaf	*Staphylococcus aureus*	≤0.01	Controlled the growth of methicillin-resistant *S. aureus* biofilm	Vijayakumar et al. [55]
*Aloe barbadensis*	Leaf	*Escherichia coli*, *Pseudomonas aeruginosa* and *Staphylococcus aureus*	2.2–2.4	Significant antibacterial activity against extended spectrum β-lactamases (ESBL) positive *E. coli*, *P. aeruginosa*, and methicillin resistant *S. aureus* (MRSA) clinical isolates	Ali et al. [131]
*Anchusa italica*	Flower	*Bacillus megaterium*, *Stapphylococcus aureus*, *Escherichia coli* and *Salmonella typhimurium*	0.016–0.032	Showed antimicrobial activity against Gram positive and Gram negative bacteria decreased with increasing the heat treating temperature	Azizi et al. [6]
*Trifolium pratense*	Flower	*Escherichia coli*, *Pseudomonas aeruginosa*, *and Staphylococcus aureus*	–	Exhibited high activity against standard and clinical strain of Gram-positive and Gram-negative bacteria	Dobrucka and Długaszewska [15]
*Rosa canina*	Fruit	*Listeria monocytogenes*, *Staphylococcus aureus* and *Escherichia coli*	0.5–1	Relatively good antibacterial activity against Gram positive and Gram negative bacteria	Jafarirad et al. [57]
*Azadirachta indica*	Leaf	*Klebsiella aerogenes* and *Staphylococcus aureus*	0.1–1	Showed significant antibacterial activity against *K. aerogenes* and *S. aureus*	Madan et al. [36]
*Limonia acidissima*	Leaf	*Mycobacterium tuberculosis*	0.0125	Control the growth of *M. tuberculosis*	Patil and Taranath [59]
*Carica papaya*	Milk	*Pseudomonas aeruginosa*, *Staphylococcus aureus*, *Klebsiella aerogenes* and *Pseudomonas desmolyticum*	0.2–0.4	Showed significant antibacterial activity against bacterial stains	Sharma [60]
*Jacaranda mimosifolia*	Flower	*Escherichia coli* and *Enterococcus faecium*	0.1	Enhanced antibacterial activity against pathogenic strains	Sharma et al. [136]
*Boswellia ovalifoliolata*	Bark	*Sphingobacterium thalpophilum*, Uncultured organism clone, *Ochrobactrum* sp., Uncultured *Achromobacter* sp., Uncultured bacterium clone, *Sphingobacterium* sp., *Acinetobacter* sp., Uncultured soil bacterium, *Ochrobactrum* sp., Uncultured bacterium	-	Showed good antibacterial activity at 170 ppm compared to 50 and 100 ppm	Supraja et al. [61]
*Heritiera fomes*	Bark and Leaf	*Shigella flexneri*	0.1	Displayed positive inhibition activity against *S. flexneri*	Thatoi et al. [16]
*Sonneratia apetala*					
*Citrullus colocynthis*	Fruit, Seed and Pulp	*Bacillus subtilis*, Methicillin-resistant *Staphylococcus aureus*, *Pseudomonas aeruginosa* and *Escherichia coli*	–	Inhibited the growth of medically significant pathogenic Gram positive and Gram negative bacteria	Azizi et al. [80]
*Cochlospermum religiosum*	Leaf	*Bacillus subtilis*, *Staphylococcus aureus*, *Pseudomonas aeruginosa* and *Escherichia coli*	0.004–0.312	Showed significant inhibition against Gram positive and Gram negative bacteria	Mahendra et al. [82]
*Pongamia pinnata*	Seed	*Bacillus licheniformis*, *Pseudomonas aeruginosa*, *Vibrio parahaemolyticus*	0.025	Effectively inhibited Gram positive and Gram negative bacteria growth	Malaikozhundan et al. [111]
*Ceropegia candelabrum*	Leaf	*Staphylococcus aureus*, *Bacillus subtilis*, *Escherichia coli*, *Salmonella typhi*	0.1	Showed significant inhibition against Gram positive and Gram negative bacterial pathogens	Murali et al. [5]
*Passiflora caerulea*	Leaf	*Klebsiella* sp., *Streptococcus* sp., *Enterococcus* sp., and *Escherichia coli*	-	Showed very good inhibition of urinary tract infection causing microbes	Santhoshkumar et al. [100]
*Couroupita guianensis*	Leaf	*Bacillus cereus*, *Klebsiella pneumoniae*, *Escherichia coli*, *Micrococcus luteus*, *Salmonella typhi*,and *Vibrio cholerae*	0.005	Exhibited excellent dose dependent bactericidal effect against human pathogens	Sathishkumar et al. [112]
*Conyza canadensis*	Leaf	*Escherichia coli* and *Staphylococcus aureus*	0.055–0.094	Exhibited strong antibacterial activity	Ali et al. [85]
*Catharanthus roseus*	Leaf	*Staphylococcus aureus*, *Streptococcus pyogenes*, *Bacillus cereus*, *Pseudomonas aeruginosa*, *Proteus mirabilis* and *Escherichia coli*	1.5	Displayed good antibacterial activity against pathogenic bacteria	Gupta et al. [113]
*Coccinia abyssinica*	Tuber	*Bacillus coagulans*, *Staphylococcus aureus*, *Shigella dysenteriae*, *Salmonella typhimurium* and *Sphingomonas paucimobilis*	0.001–0.005	Showed effective growth inhibition activity against Gram negative and Gram positive bacteria	Safawo et al. [117]
*Bauhinia tomentosa*	Leaf	*Bacillus subtilis*, *Staphylococcus aureus*, *Escherichia coli* and *Pseudomonas aeruginosa*	–	Exhibited better antibacterial activity against Gram negative bacteria than Gram positive bacteria	Sharmila et al. [30]
*Costus pictus*	Leaf	*Staphylococcus aureus*, *Bacillus subtilis*, *Escherichia coli*, *Salmonella paratyphi*	0.1	Exhibited strong antimicrobial behavior against bacterial species	Suresh et al. [66]
*Atalantia monophylla*	Leaf	*Bacillus subtilis*, *Bacillus cereus*, *Staphylococcus aureus*, *Escherichia coli*, *Pseudomonas aeruginosa* and *Klebsiella pnemoniae*	–	Showed antimicrobial potential against pathogenic bacteria	Vijayakumar et al. [103]
*Berberis aristata*	Leaf	*Escherichia coli*, *Staphylococcus aureus*, *Klebsiella pneumoniae*, *Bacillus subtilis*, *Bacillus cereus* and *Serratia marcescens*	0.064–0.256	Displayed antibacterial activity against urinary tract infection causing pathogens	Chandra et al. [118]
*Laurus nobilis*	Leaf	*Escherichia coli*	1.2	Proved as an effective antibacterial agent against *E. coli*	Chemingui et al. [92]
*Juglans regia*	Leaf	*Escherichia coli*, *Pseudomonas aeruginosa* and *Acinetobacter baumannii*	0.2	Exerted bactericidal property on resistant strains	Darvishi et al. [119]
*Populus ciliata*	Leaf	*Escherichia coli*, *Pseudomonas aeruginosa*, *Klebsiella pneumonia*, *Staphylococcus aureus and Streptococcus pyogene*	–	Showed significant antibacterial potential on test pathogens	Hafeez et al. [90]
*Pandanus odorifer*	Leaf	*Bacillus subtilis*, *Escherichia coli*	0.05	Showed significant antibacterial potential on test pathogens	Hussain et al. [121]
*Dolichos lablab*	Leaf	*Bacillus pumilus* and *Sphingomonas paucimobilis*	5	Showed a bactericidal activity for pathogenic Gram positive and Gram negative bacteria	Kahsay et al. [122]
*Trianthema portulacastrum*	Plant	*Staphylococcus aureus* and *Escherichia coli*	–	Showed significant antibacterial property	Khan et al. [39]
*Mentha pulegium*	Leaf	*Staphylococcus aureus* and *Escherichia coli*	0.2	Exhibited significant antimicrobial potential on some food-borne pathogens	Rad et al. [91]
*Annona squamosa*	Leaf	*Escherichia coli*, *Bacillus subtilis*, *Staphylococcus aureus*, *Pseudomonas aeruginosa*, *Enterococcus faecium*	0.006–0.012	Synergetic antibacterial potential against wound/burn infection causing bacteria	Ruddaraju et al. [69]
*Artabotrys hexapetalu*	Leaf	*Streptococcus* and *Serratia*	–	Showed better antibacterial performance against Gram positive and Gram negative bacteria	Shanavas et al. [68]
*Bambusa vulgaris*					
*Tecoma castanifolia*	Leaf	*Bacillus subtilis*, *Staphylococcus aureus*, *Escherichia coli*, *Pseudomonas aeruginosa*	0.075–0.1	Excellent antibacterial activity against Gram positive and Gram negative bacteria	Sharmila et al. [132]
*Albizia lebbeck*	Bark	*Bacillus cereus*, *Staphylococcus aureus*, *Escherichia coli*, *Klebsiella pneumoniae*,and *Salmonella typhi*	35.5	Strong antibacterial potential against Gram-negative and Gram-positive bacterial pathogens	Umar et al. [26]
*Lycopersicon esculentum*	Leaf	*Enterococcus faecalis* and *Proteus vulgaris*	0.008–0.01	A notable reduction in bacterial growth was observed	Vijayakumar et al. [137]
*Costus igneus*	Leaf	*Streptococcus mutans*, *Lysinibacillus fusiformis*, *Proteus vulgaris*,and *Vibrio parahaemolyticus*	0.04–0.07	Showed promising antibacterial activity against targeted pathogenic bacteria	Vinotha et al. [33]
*Mucuna pruriens*	Seed	*Bacillus subtilis*	0.02	Showed concentration dependent inhibition of the growth of *B. subtilis*	Agarwal et al. [27]
*Sambucus ebulus*	Leaf	*Bacillus cereus*, *Staphylococcus aureus*,and *Escherichia coli*	0.1	Exhibited antibacterial activity over all three bacteria	Alamdari et al. [124]
*Cinnamomum verum*	Bark	*Escherichia coli* and *Staphylococcus aureus*	0.062–0.125	Inhibited the growth of harmful pathogens	Ansari et al. [2]
*Citrus sinensis*	Fruit Peel	*Escherichia coli* and *Staphylococcus aureus*	0.020–0.040	Showed stronger antibacterial activity	Gao et al. [19]
*Mussaenda frondosa*	Leaf, Stem and Leaf-derived callus	*Staphylococcus aureus*, *Bacillus subtilis*, *Escherichia coli* and *Pseudomonas aeruginosa*	0.019–0.185	Showed inhibition against bacterial strains	Jayappa et al. [97]
*Cratoxylum formosum*	Leaf	*Bacillus subtilis*, *Staphylococcus epidermidis*, *Escherichia coli*	5	Inhibited Gram positive and Gram negative bacterial growth	Jevapatarakul et al. [105]
*Aegle marmelos*	Juice	*Staphylococcus aureus*, *Bacillus cereus*, *Micrococcus luteus*, *Escherichia coli*, *Klebsiella pneumonia*, *Enterobacter aerogenes*, *Pseudomonas fluorescens*, *Pseudomonas aeruginosa* and *Salmonella enteritidis*	3.84–8.65	Showed good bactericidal activity	Mallikarjunaswamy et al. [98]
*Cassia fistula* and *Melia azadarach*	Leaf	*Escherichia coli* and *Staphylococcus aureus*	0.05	Showed strong antimicrobial activity against clinical pathogens	Naseer et al. [126]
*Beta vulgaris*	Plant	*Escherichia coli* and *Staphylococcus aureus*	–	Shown antibacterial activity both Gram negative and Gram positive bacteria	Pillai et al. [25]
*Cinnamomum tamala*					
*Cinnamomum verum*					
*Brassica oleracea*					
*Zea mays*	Husk	*Enterococcus faecalis*	–	Excellent antibacterial activity against *E. faecalis* compared to zinc oxide synthesized without plant extract and commercial zinc oxide	Quek et al. [31]
*Artocarpus heterophyllus*	Peel				
*Punica granatum*					
*Crotalaria verrucosa*	Leaf	*Escherichia coli*, *Staphylococcus aureus*, *Proteus vulgaris*,and *Klebsiella pneumonia*	0.1	Exhibited significant antibacterial potentiality against Gram positive and Gram negative pathogenic bacteria	Sana et al. [71]
*Aloe vera*	Leaf	*Bacillus subtilis*, *Staphylococcus aureus* and *Escherichia coli*	0.195–3.125	Showed antibacterial activity against pathogenic bacteria	Sharma et al. [127]
*Thlaspi arvense*	Plant	*Escherichia coli*	0.015	Exhibited a significant antibacterial activity against Gram negative *E. coli*	Ullah et al. [108]
*Myristica fragrans*	Fruit	*Klebsiella pneumoniae*, *Escherichia coli*, *Pseudomonas aeruginosa*,and *Staphylococcus aureus*	1	Shown successful capacity against bacterial strains	Faisal et al. [11]
**ANTIFUNGAL ACTIVITY**					
*Azadirachta indica*	Leaf	*Candida albicans* and *Candida tropicalis*	0.006–0.05	Showed significant inhibition against fungal strains in a dose-dependent manner	Elumalai and Velmurugan [13]
*Vitex trifolia*	Leaf	*Candida albicans* and *Candida tropicalis*	0.006–0.05	Excellent antifungal activity against human pathogenic fungi	Elumalai et al. [72]
*Boswellia ovalifoliolata*	Bark	*Meyerozyma caribbica*, *Aspergillus parvisclerotigenus*, *Meyerozyma guilliermondii*, *Rhizopus oryzae*, Uncultured fungus clone, *Aspergillus oryzae*, *Trichoderma asperellum*	–	Showed good antifungal activity at 170 ppm compared to 50 and 100 ppm	Supraja et al. [61]
*Pongamia pinnata*	Seed	*Candida albicans*	0.05	Effectively inhibited the biofilm formation of *C. albicans*	Malaikozhundan et al. [111]
*Ziziphus nummularia*	Leaf	*Candida albicans*, *Candida glabrata* and *Cryptococcus neoformans*	1.25–10	Showed very good antifungal activity against clinical isolates	Padalia and Chanda [7]
*Nyctanthes arbor-tristis*	Flower	*Alternaria alternata*, *Aspergillus niger*, *Botrytis cinerea*, *Fusarium oxysporum* and *Penicillium expansum*	0.016	Showed good antifungal potential against fungal phytopathogens	Jamdagni et al. [32]
*Costus pictus*	Leaf	*Aspergillus niger* and *Candida albicans*	0.1	Exhibited strong antimicrobial behavior against fungal species	Suresh et al. [66]
*Atalantia monophylla*	Leaf	*Candida albicans* and *Aspergillus niger*	–	Showed antimicrobial potential against pathogenic fungi	Vijayakumar et al. [103]
*Trianthema portulacastrum*	Plant	*Aspergillus niger*, *Aspergillus flavus* and *Aspergillus fumigatus*	0.1	Showed significant antifungal property	Khan et al. [39]
*Lycopersicon esculentum*	Leaf	*Candida albicans*	0.013	A notable reduction in fungal growth was observed	Vijayakumar et al. [137]
*Eucalyptus globules*	Leaf	*Alternaria mali*, *Botryosphaeria dothidea* and *Diplodia seriata*	–	Showed considerable fungicidal property against phytopathogenic fungi	Ahmad et al. [94]
*Citrus sinensis*	Peel	*Botrytis cinerea*	0.2	Showed stronger antifungal activity against *B. cinerea*	Gao et al. [19]
*Beta vulgaris*	Plant	*Candida albicans* and *Aspergillus niger*	–	Shown activity against the fungal strains	Pillai et al. [25]
*Cinnamomum tamala*					
*Cinnamomum verum*					
*Brassica oleracea*					

* Range of MIC concentration depicts the changes in the concentrations between the test pathogens.

**Table 3:** Antioxidant activity of plant-mediated zinc oxide nanoparticles.

Plant Name	Description	Concentration	Maximum Activity	Results	Reference
*Polygala tenuifolia*	Root	1 mg·mL^−1^	45.47%	Moderate antioxidant activity by scavenging DPPH free radical	Nagajyothi et al. [17]
*Cassia fistula*	Leaf	2853 µg·mL^−1^	50%	Inhibiting DPPH free radical scavenging activity	Suresh et al. [76]
*Artocarpus gomezianus*	Fruit	10.8 mg·mL^−1^	50%	Inhibiting DPPH free radical scavenging activity	Suresh et al. [77]
*Rosa canina*	Fruit	0.2 mg·mL^−1^	>90%	DPPH free radical scavenging attribute	Jafarirad et al. [57]
*Azadirachta indica*	Leaf	8355 μg·mL^−1^	92%	Inhibiting DPPH free radical scavenging activity	Madan et al. [36]
*Heritiera fomes* and *Sonneratia apetala*	Bark and leaf	53.64 μg·mL^−1^	50%	Strong DPPH free radical scavenging potential	Thatoi et al. [16]
*Citrullus colocynthis*	Fruit, seed and pulp	0.22 mg·mL^−1^ (Fruit), 0.29 mg·mL^−1^ (Seed) and 0.26 mg·mL^−1^ (Pulp)	50%	Inhibiting DPPH free radical scavenging activity	Azizi et al. [80]
*Ceropegia candelabrum*	Leaf	95.09 μg·mL^−1^	55.43%	DPPH free radical scavenging activity	Murali et al. [5]
*Azadirachta indica*, *Hibiscus rosa-sinensis*, *Murraya koenigii*, *Moringa oleifera* and *Tamarindus indica*	Leaf	11.03–31.51 µg·mL^−1^ (ABTS), 11.49–37.8 µg·mL^−1^ (DPPH), 23.31–45.9 µg·mL^−1^ (hydroxyl), 24.4–53.2 µg·mL^−1^ (superoxide) and 31.4–58.4 µg·mL^−1^ (hydrogen peroxide)	50%	Inhibition of ABTS, DPPH, hydroxyl, superoxide and hydrogen peroxide radical scavenging activities	Rehana et al. [22]
*Eucalyptus globulus*	Leaf	46.62 μg·mL^−1^	82%	DPPH free radical scavenging inhibition	Siripireddy and Mandal [83]
*Andrographis paniculata*	Leaf	500 μg·mL^−1^	61.32%	DPPH free radical scavenging inhibition	Rajakumar et al. [34]
*Mangifera indica*	Leaf	30 μg·mL^−1^	65%	DPPH free radical scavenging activity	Rajeshkumar et al. [65]
*Coccinia abyssinica*	Tuber	127.74 μg·mL^−1^	50%	DPPH free radical scavenging activity	Safawo et al. [117]
*Kalanchoe pinnata*	Leaf	700 μg·mL^−1^	50%	Reduce DPPH free radical scavenging capacity	Agarwal and Shanmugam [21]
*Berberis aristata*	Leaf	3.55 μg·mL^−1^	50%	DPPH free radical scavenging activity	Chandra et al. [118]
*Trianthema portulacastrum*	Plant	500 μg·mL^−1^	75%	Efficient DPPH free radical inhibition	Khan et al. [39]
*Tecoma castanifolia*	Leaf	100 μg·mL^−1^	67%	DPPH free radical scavenging activity	Sharmila et al. [132]
*Scutellaria baicalensis*	Root	1000 µg·mL^−1^	56.11%	Scavenging DPPH free radicals	Tettey and Shin [70]
*Albizia lebbeck*	Stem bark	48.5 µg·mL^−1^	50%	Showed the concentration dependent effect in hydrogen peroxide (H_2_O_2_) free radical scavenging activity	Umar et al. [26]
*Costus igneus*	Leaf	100 μg·mL^−1^	75%	DPPH free radical scavenging activity	Vinotha et al. [33]
*Sambucus ebulus*	Leaf	43 µg·mL^−1^	50%	Exhibited hydrogen peroxide (H_2_O_2_) free radical scavenging activity	Alamdari et al. [124]
*Mussaenda frondosa*	Leaf, stem and leaf-derived callus	824 µg·mL^−1^ (Leaf), 752 µg·mL^−1^ (Stem) and 857 µg·mL^−1^ (Callus)	50%	Quenching the DPPH free radical scavenging	Jayappa et al. [97]
*Aegle marmelos*	Juice	5.75–6.78 mg·mL^−1^ (DPPH), 4.45–5.05 mg·mL^−1^ (ABTS) and 7.86–9.05 mg·mL^−1^ (Superoxide)	50%	ABTS cation radical, DPPH free radical, and superoxide anion radical scavenging activities	Mallikarjunaswamy et al. [98]
*Zea mays*, *Artocarpus heterophyllus* and *Punica granatum*	Husk (*Z. mays*) and peel (*A. heterophyllus* and *P. granatum*)	395.2 µg·mL^−1^ (*P. granatum*)	50%	Inhibitory of DPPH radical scavenger	Quek et al. [31]
*Myristica fragrans*	Fruit	400 μg·mL^−1^	82.12 TEAC (ABTS); 66.3% FRSA (DPPH); 71.1 μg AAE/mg (TAC); 63.41 μg AAE/mg (TRP)	Excellent free radical scavenging activities (ABTS, DPPH, TAC and TRP)	Faisal et al. [11]

Note: ABTS—2,2′-azino-bis(3-ethylbenzothiazoline-6-sulphonic acid) diammonium salt, DPPH—2,2-diphenyl-1-picrylhydrazyl, TAC—total antioxidant capacity, TRP—total reduction power, and FRSA—free radical scavenging assay.

**Table 4:** Antidiabetic activity of plant-mediated zinc oxide nanoparticles.

Plant Name	Description	Concentration	Activity (IC_50_Value) * (mg mL^−1^)	Results	Reference
*Hibiscus subdariffa*	Leaf	8 mg·kg^−1^ of body weight	–	Streptozotocin (STZ: 100 mg/kg of body weight) induced diabetes was cured by intraperitoneal injection of zinc oxide in mice	Bala et al. [35]
*Heritiera fomes* (HF)and *Sonneratia apetala* (SA)	Bark and leaf	100 μL	0.33 (HF) and 0.39 (SA)	Exhibited better anti-diabetic activity in terms of α-amylase inhibition activity	Thatoi et al. [16]
*Azadirachta indica*, *Hibiscus rosa-sinensis*, *Murraya koenigii*, *Moringa oleifera* and *Tamarindus indica*	Leaf	100–1.52 µg·mL^−1^	α-amylase: 0.025–0.05 α-glucosidase: 0.012–0.05	Exhibited higher α-amylase and α-glucosidase inhibition activity	Rehana et al. [22]
*Vaccinium arctostaphylos*	Fruit	–	–	Exhibited great treating efficacy on alloxan-diabetic rats compared to chemically synthesized zinc oxide	Bayrami et al. [63]
*Andrographis paniculata*	Leaf	100 μL	0.12	Exhibited better anti-diabetic activity in terms of exhibiting moderate α-amylase inhibitory activity	Rajakumar et al. [34]
*Costus igneus*	Leaf	100 μg·mL^−1^	–	Increased the percentage of α-amylase and α-glucosidase inhibition with increased concentration of nanoparticles	Vinotha et al. [33]
*Mussaenda frondosa*	Leaf, stem and leaf-derived callus	20 μL	α-amylase: 0.014- 0.055 α-glucosidase: 0.014–0.035	Exhibited on par α-amylase inhibitory activity and α-glucosidase inhibitory activity	Jayappa et al. [97]
*Myristica fragrans*	Fruit	400 μg·mL^−1^	–	Excellent α-amylase and α-glucosidase inhibition activity	Faisal et al. [11]

* Range of the IC_50_ value depicts the changes in between the methods employed.

**Table 5:** Anticancer activity of plant-mediated zinc oxide nanoparticles.

Plant Name	Description	Cell Lines Used	Activity (IC_50_Value)	Results	Reference
*Anchusa italica*	Flower	Vero cells	142 μg·mL^−1^	Showed concentration-dependent cytotoxicity on the growth of Vero cells	Azizi et al. [6]
*Rosa canina*	Fruit	Alveolar adenocarcinoma (A549) cells	>0.1 mg·mL^−1^	Exhibited dose-dependent toxicity to A549 cells	Jafarirad et al. [57]
*Citrullus colocynthis*	Fruit, seed and pulp	3T3 cells	0.258 mg·mL^−1^ (Fruit), 0.160 mg·mL^−1^ (Seed) and 0.210 mg·mL^−1^ (Pulp)	Showed a dose dependent toxicity on the growth of 3T3 cells with non-toxic effect of concentration below 0.26 mg/mL	Azizi et al. [80]
*Pongamia pinnata*	Seed	Human MCF-7 breast cancer cell lines	50 μg·mL^−1^	More successful in control of human MCF-7 breast cancer cells compared to the seed extract and bulk zinc oxide (positive control)	Malaikozhundan et al. [111]
*Ziziphus nummularia*	Leaf	HeLa cancer cell lines	50 and 200 μg·mL^−1^	Showed potent dose-dependent cytotoxic effect against HeLa cancer cell lines	Padalia and Chanda [7]
*Mangifera indica*	Leaf	Lung cancer A549 cell lines	25 μg·mL^−1^	Significant cytotoxic effect against lung cancer A549 cell lines	Rajeshkumar et al. [65]
*Costus pictus*	Leaf	Daltons lymphoma ascites (DLA) cells	50 µg·mL^−1^	Exhibited strong anticancer behavior against DLA bearing mice cell lines	Suresh et al. [66]
*Anacardium occidentale*	Leaf	Human normal fibroblast cell line (Hu02) and human pancreatic cancer cell lines (Panc-1 and AsPC-1)	40 μM (Panc-1) and 30 μM (AsPC-1)	Exhibited the concentration-dependent cytotoxicity against human pancreatic cancer cell lines	Zhao et al. [88]
*Kalanchoe pinnata*	Leaf	Murine macrophage RAW 264.7 cells	–	Exhibited no significant cytotoxicity up to 1 mg/mL in RAW 264.7 cells	Agarwal and Shanmugam [21]
*Gracilaria edulis*	Aqueous extract	Cervical carcinoma cells (SiHa cells)	35 μg·mL^−1^	Exhibited cytotoxic effect against SiHa cells in a dose dependent manner	Asik et al. [89]
*Juglans regia*	Leaf	Human skin fibroblasts	200 μg·mL^−1^	Have less cytotoxicity than chemical zinc oxide nanoparticles	Darvishi et al. [119]
*Cucurbita pepo*	Leaf	Mammalian osteoblast-like MG63 cells	20 ppm	Induced cytotoxicity that affected the proliferation of MG63 cells in the concentration dependent manner	Hu et al. [120]
*Pandanus odorifer*	Leaf	Breast cancer (MCF-7), liver cancer (HepG2), and lung cancer (A-549) cells	100 μg·mL^−1^	Apoptotic and necrosis effect on MCF-7, HepG2, and A549 cancer cell lines	Hussain et al. [121]
*Trianthema portulacastrum*	Plant	Mouse pre-osteoblast cell line (MC3T3-E1)	–	Showed no toxic effect and the cells were found viable	Khan et al. [39]
*Cucumis melo inodorus*	Rough shell	Human (Michigan Cancer Foundation-7 [MCF7]) and murine (TUBO) breast cancer cell lines	40 µg·mL^−1^ (MCF7); 20 µg·mL^−1^ (TUBO)	Found as a powerful apoptosis inducer in breast cancer cells in human cell line (MCF7) and murine (TUBO cell line and cancer model)	Mahdizadeh et al. [101]
*Artocarpus heterophyllus*	Leaf	Human colon cancer HCT-116 cell lines	20 μg·mL^−1^	Showed excellent cytotoxic effect against human colon cancer HCT-116 cell lines	Majeed et al. [93]
*Hyssops officinalis*	Plant	MDA-MB231 breast cancer cell line	125 μg·mL^−1^	Inhibitory effects on the growth of breast cancer cells and induction of cytotoxicity depending on nanoparticle concentration and time of exposure	Rahimi Kalateh Shah Mohammad et al. [102]
*Annona squamosa*	Leaf	Cervical cancer cells (HeLa cell lines)	50 μg·mL^−1^	Anticancer activity against HeLa cell lines in a dose dependent pattern with a defensive prospect towards mammalian (HEK-293) cells	Ruddaraju et al. [69]
*Tecoma castanifolia*	Leaf	Human lung carcinoma cells (A549)	65 μg·mL^−1^	Conferred better cytotoxic effects on proliferation of A549 cell line	Sharmila et al. [132]
*Scutellaria baicalensis*	Root	HeLa cells (Human cervical cancer cell line) and RAW 264.7 murine macrophage cells	1000 µg·mL^−1^	Showed dose-dependent antiproliferative activity against the growth of HeLa cells and no toxicity on RAW 264.7 macrophages (normal immune system cells)	Tettey and Shin [70]
*Albizia lebbeck*	Stem bark	Human breast cancer cell lines (MDA-MB 231 and MCF-7)	Cytotoxicity: 100 µg·mL^−1^ (MDA-MB 231) and 5 µg·mL^−1^ (MCF-7); Proliferation: 100 µg·mL^−1^ (MDA-MB 231 and MCF-7)	Inhibited the cell viability and cell number (proliferation) of MDA-MB 231 and MCF-7 cells in concentration dependent manner	Umar et al. [26]
*Lycopersicon esculentum*	Leaf	Murine macrophage cells (RAW 264.7) and Human cervical cancer (HeLa) cells	100 µg·mL^−1^	Zinc oxide nanoparticles were non-toxic to macrophage cells, as no alterations in viability. Treatment of HeLa cells with zinc oxide nanoparticles induced cell growth retardation, cell clumping, cell bursting, and loss of membrane stability and they prevented the proliferation of HeLa cells	Vijayakumar et al. [137]
*Rehmanniae radix*	Plant	Bone cancer cell line MG-63	30 μg·mL^−1^	Exhibited strong anticancer activity and inducing apoptosis on MG-63 cells via stimulating increased generation of ROS	Cheng et al. [104]
*Citrus sinensis*	Peel	Human umbilical vein endothelial cells (HUVECs)	Below 25 mg·L^−1^	Cytotoxicity towards HUVECs exhibited when the concentration exceeded 12.5 mg L^−1^	Gao et al. [19]
*Mussaenda frondosa*	Leaf, stem and leaf-derived callus	Human lung adenocarcinoma cells (A549)	67.75 µg·mL^−1^ (Callus) and 85.66 µg·mL^−1^ (Stem)	Exhibited on par cytotoxic activity on A549 cells in a dose-dependent action	Jayappa et al. [97]
*Hyssopus officinalis*	Leaf	Human prostate cancer (PC3) cells	8.07 µg·mL^−1^ (24 h) and 5 µg·mL^−1^ (48 h)	Demonstrated the dose-dependent cytotoxicity effect and induced apoptosis on PC3 cells	Rahimi Kalateh Shah Mohammad et al. [107]
*Crotalaria verrucosa*	Leaf	HeLa and DU145 cell lines	7.07 µg·mL^−1^ (HeLa); 6.30 µg·mL^−1^ (DU145)	Exhibited the dose-dependent inhibition curve with IC_50_ value of 7.07 µg/mL and 6.30 µg/mL in HeLa and DU145 cells, respectively	Sana et al. [71]
*Deverra tortuosa*	Plant	Human colorectal epithelial adenocarcinoma (Caco-2), human lung epithelial carcinoma (A549) and normal human lung fibroblast cell line (WI38)	83.47 μg·mL^−1^ (A549), 50.81 μg·mL^−1^ (Caco-2) and 434.60 μg·mL^−1^ (WI38)	Exhibited the profound selective concentration dependent cytotoxic effect on Caco-2 and A549 cancer cell lines with appreciable lower cytotoxic activity on normal WI38 cells	Selim et al. [99]
*Euphorbia fischeriana*	Root	Lung cancer (A549) cells	14.5 µg·mL^−1^	Induced cytotoxicity and also activated apoptosis during increased ROS formation, decreased mitochondrial membrane potential, inhibited cell migration, altered AO/EtBr staining and induced pro-apoptotic and inhibited anti-apoptotic protein	Zhang et al. [129]
*Myristica fragrans*	Fruit	*Streptomyces* 85E strain for protein kinase inhibition capability	5 mg·mL^−1^	Clear zones were observed against *Streptomyces* 85E strain which used to elucidate the protein kinase inhibition capability	Faisal et al. [11]
*Raphanus sativus*	Leaf	Lung cancer cell line (A549)	40 μg·mL^−1^	Showed a better anticancer activity by reducing cell viability	Umamaheswari et al. [29]

**Table 6:** Anti-inflammatory activity of plant-mediated green synthesized zinc oxide nanoparticles.

Plant Name	Description	Assay/Model	Activity (IC_50_Value)	Results	Reference
*Polygala tenuifolia*	Root	LPS-stimulated RAW 264.7 murine macrophage cells	1 mg·mL^−1^	Showed anti-inflammatory activity by suppressing the LPS-induced mRNA and protein expressions of iNOS, COX-2, and anti-inflammatory cytokines in LPS-stimulated RAW 264.7 murine macrophage cells	Nagajyothi et al. [17]
*Heritiera fomes* (HF) and *Sonneratia apetala* (SA)	Bark and leaf	Inhibition of protein denaturationin vitroassay	72.35 μg·mL^−1^ (HF) and 63.29 μg·mL^−1^ (SA)	Anti-inflammation activity inhibiting protein (heat induced albumin) denaturation	Thatoi et al. [16]
*Andrographis paniculata*	Leaf	Inhibition of protein denaturationin vitroassay	66.78 μg·mL^−1^	Anti-inflammatory activity by inhibiting protein denaturation	Rajakumar et al. [34]
*Kalanchoe pinnata*	Leaf	LPS-induced Murine Raw 264.7 cell lines; Detection of the mRNA expressions of TNF-α, IL-1β, IL-6, and COX-2	–	Reduced the expression of pro-inflammatory cytokines, attenuated the release of IL-1β, IL-6, and TNF-α by inhibiting mRNA expression, inhibited the gene expression of COX-2 enzyme and suppressed NO production	Agarwal and Shanmugam [21]
*Hyssops officinalis*	Plant	Reduction of mouse paw edema	5 mg·kg^−1^	Reduction of inflammation by significantly reducing the thickness of mouse paw edema	Mohammad et al. [102]
*Mussaenda frondosa*	Leaf, stem & leaf-derived callus	Human red blood cells membrane stabilization method	500 µg·mL^−1^	Exhibited varying degrees of human RBCs membrane and lysosomal membrane stabilizing activity in a dose-dependent manner	Jayappa et al. [97]
*Vernonia amygdalina*	Leaf	Swiss Albino male mice	2.5, 5, and 7.5 mg·kg^−1^	Exhibited the potent anti-inflammatory activity against carrageenan induced-inflammation in mice	Liu et al. [125]

**Table 7:** Photocatalytic activity of the plant-mediated zinc oxide nanoparticles.

Plant Name	Dye Degraded	Solar Irradiation Time	pH Range	Degradation Efficiency (%)	Reference
*Phyllanthus niruri*	Methylene blue (MB)	30 min	–	99%	Anbuvannan et al. [53]
*Anisochilus carnosus*	Methylene blue (MB)	90 min	–	100%	Anbuvannan et al. [54]
*Vitex trifolia*	Methylene blue (MB)	90 min	–	92.13%	Elumalai et al. [72]
*Plectranthus amboinicus*	Methyl red (MR)	180 min	–	92.45%	Fu and Fu [73]
*Allium sativum*, *Allium cepa* and *Petroselinum crispum*	Methylene blue	180 min	–	>90%	Stan et al. [74]
*Cassia fistula*	Methylene blue	120 min	7	90% (for 5 ppm)	Suresh et al. [76]
*Artocarpus gomezianus*	Methylene blue (MB)	120 min	10	100% (for 5 ppm, sun light); 65% (for 5 ppm, UV light)	Suresh et al. [77]
*Corymbia citriodora*	Methylene blue	90 min	–	83.45%	Zheng et al. [78]
*Mimosa pudica*	Methylene blue	120 min	–	90%	Fatimah et al. [110]
*Coffea arabica*	Methylene blue	120 min	–	98%	
*Carissa edulis*	Congo red	130 min	–	97%	Fowsiya et al. [56]
*Nephelium lappaceum*	Methyl orange (MO)	120 min	7.01	83.99%	Karnan and Selvakumar [58]
*Azadirachta indica*	Methylene blue (MB)	120 min	–	>80%	Madan et al. [36]
*Terminalia chebula*	Rhodamine B (RhB)	5 h	–	70% (for 5 ppm)	Rana et al. [79]
*Carica papaya*	Alizarin Red-S	120 min	–	99%	Sharma [60]
*Sedum alfredii*	2-chlorophenol (2-CP)	120 min	6.3	96.93%	Wang et al. [133]
*Camellia sinensis*	Methylene blue (MB)	240 min	–	100%	Nava et al. [62]
*Eucalyptus globulus*	Methylene blue (MB) and Methyl orange (MO)	50 min (MB) and 1 h (MO)	–	98.2% (MB) and 96.6% (MO)	Siripireddy and Mandal [83]
*Acacia senegal*	Direct blue 129 (DB129)	105 min	–	95%	Taghavi Fardood et al. [84]
*Conyza canadensis*	Methylene blue (MB) and Methyl orange (MO)	45 min (MO) and 20 min (MB)	–	94.5% (MO) and 85.3% (MB)	Ali et al. [85]
*Garcinia mangostana*	Malachite green	180 min	–	99%	Aminuzzaman et al. [86]
*Citrus sinensis*	Methylene blue (MB)	120 min	–	83%	Luque et al. [64]
*Ferulago angulata*	Rhodamine B (RhB)	150 min	–	93%	Mehr et al. [115]
*Laurus nobilis*	Remazol Brilliant Red F3B (Reactive Red 180, RR180)	45 min	Around 6.8	99%	Chemingui et al. [92]
*Dolichos lablab*	Methylene blue (MB), Rhodamine B (RhB) and Orange II (OII)	210 min	11 (MB), 9 (RhB) and 5 (OII)	80% (MB), 95% (RhB), and 66% (OII)	Kahsay et al. [122]
*Trianthema portulacastrum*	Synozol navy blue K-BF	150min	–	91%	Khan et al. [39]
*Abelmoschus esculentus*	Methylene blue (MB) and Rhodamine B (RhB)	60 min (MB) and 50 min (RhB)	∼7	100%	Prasad et al. [123]
*Artabotrys hexapetalu* (AH) and *Bambusa vulgaris* (BV)	Rhodamine B (RhB)	180 min	Neutral	92% (AH) and 88% (BV)	Shanavas et al. [68]
*Musa acuminata*	Methylene blue (MB)	7 h	12	98.13%	Abdullah et al. [40]
*Sambucus ebulus*	Methylene blue	200 min	–	80%	Alamdari et al. [124]
*Ziziphus jujuba*	Methylene blue (MB) and Eriochrome black-T (ECBT)	5 h	–	92% (MB) and 86% (ECBT)	Golmohammadi et al. [96]
*Mussaenda frondosa*	Methylene blue	100 min (Leaf), 100 min (Stem), and 120 min (Callus)	7	30% (Leaf), 30% (Stem) and 90% (Callus)	Jayappa et al. [97]
*Aegle marmelos*	Methylene blue (MB)	35 min	–	96%	Mallikarjunaswamy et al. [98]
Quince	Methylene blue	2 h	Normal	80%	Moghaddas et al. [141]
*Syzygium cumini*	Rhodamine B (RhB)	100 min	9	98%	Rafique et al. [106]
*Aloe vera*	Methyl orange (MO)	140–160 min	–	95%	Sharma et al. [127]
*Thlaspi arvense*	Methylene blue (MB)	2 h	–	100%	Ullah et al. [108]
*Calliandra haematocephala*	Methylene blue (MB)	270 min	–	88%	Vinayagam et al. [128]
*Myristica fragrans*	Methylene blue	140 min	–	88%	Faisal et al. [11]
*Bridelia retusa*	Rhodamine B	165 min	–	Upto 94.74%	Vinayagam et al. [130]

## Data Availability

The data presented in this study are available in this manuscript.
